# Serum neurofilament light chain as a prognostic marker of all-cause mortality in a national sample of US adults

**DOI:** 10.1007/s10654-024-01131-7

**Published:** 2024-05-21

**Authors:** May A. Beydoun, Nicole Noren Hooten, Michael F. Georgescu, Hind A. Beydoun, Shaker M. Eid, Marie T. Fanelli-Kuczmarski, Michele K. Evans, Alan B. Zonderman

**Affiliations:** 1 Laboratory of Epidemiology and Population Sciences, NIA/NIH/IRP, Baltimore, MD USA; 2 Department of Research Programs, Fort Belvoir Community Hospital, Fort Belvoir, VA USA; 3 Department of Medicine, Johns Hopkins Medical Institutions, Baltimore, MD USA; 4 NIH Biomedical Research Center, National Institute on Aging, IRP, 251 Bayview Blvd., Suite 100, Room #: 04B118, Baltimore, MD 21224 USA

**Keywords:** Neurofilament light chain, Mortality, Prognostic markers, Aging, Biomarker, Neurodegenerative disease, Population

## Abstract

Neurofilament light chain (NfL) is a neuron-specific structural protein released into the extracellular space, including body fluids, upon neuroaxonal damage. Despite evidence of a link in neurological disorders, few studies have examined the association of serum NfL with mortality in population-based studies. Data from the National Health and Nutrition Survey were utilized including 2,071 Non-Hispanic White, Non-Hispanic Black and Hispanic adult participants and adult participants of other ethnic groups (20–85 years) with serum NfL measurements who were followed for ≤ 6 years till 2019. We tested the association of serum NfL with mortality in the overall population and stratified by sex with the addition of potential interactive and mediating effects of cardio-metabolic risk factors and nutritional biomarkers. Elevated serum NfL levels (above median group) were associated with mortality risk compared to the below median NfL group in the overall sample (*P* = 0.010), with trends observed within each sex group (*P* < 0.10). When examining Log_e_ NfL as a continuum, one standard deviation of Log_e_ NfL was associated with an increased mortality risk (HR = 1.88, 95% CI 1.60–2.20, *P* < 0.001) in the reduced model adjusted for age, sex, race, and poverty income ratio; a finding only slightly attenuated with the adjustment of lifestyle and health-related factors. Four-way decomposition indicated that there was, among others, mediated interaction between NfL and HbA1c and a pure inconsistent mediation with 25(OH)D3 in predicting all-cause mortality, in models adjusted for all other covariates. Furthermore, urinary albumin-to-creatinine ratio interacted synergistically with NfL in relation to mortality risk both on the additive and multiplicative scales. These data indicate that elevated serum NfL levels were associated with all-cause mortality in a nationally representative sample of US adults.

**Supplementary Information:**

The online version contains supplementary material available at 10.1007/s10654-024-01131-7.

## Introduction

Neuroaxonal damage can be caused by a wide spectrum of initiating factors including ischemia, inflammation, compression, trauma and other disease processes. This injury can initiate a cascade of events leading to a variety of neurodegenerative diseases. Therefore, there is substantial interest in identifying biomarkers of neuroaxonal damage, especially early in the life course prior to the onset of disease. Recent attention has focused on blood-based measurements of neurofilament light chain (NfL) as technological advancements have enabled sensitive and high-throughput measurements of blood NfL, measured in plasma or serum. NfL is released into the extracellular space following neuroaxonal injury and can be assayed from serum, plasma (blood) and cerebrospinal fluid (CSF). Moreover, blood NfL levels correlate with CSF NfL among individuals with neurodegenerative diseases [1, 2] and are elevated in Alzheimer’s disease [3–6] and other neurodegenerative diseases [7–9].

Recently there have been several reports that have analyzed the association of plasma NfL levels with mortality in population-based studies [10–13]. Longitudinal data from the Healthy Aging in Neighborhoods of Diversity Across the Life Span (HANDLS) study found that annualized change in plasma NfL was associated with mortality only in women in this diverse cohort of middle-aged (mean age around 48 years) African American and White adults (n = 694) [10]. Furthermore, a one standard deviation elevation in plasma NfL at the baseline time point was associated with an increased risk for all-cause mortality [10]. In both men and women serum NfL levels were associated with all-cause mortality in adults aged > 65 or older from the Memory and Morbidity in Augsburg Elderly (MEMO) study [11]. In cross-sectional analyses in this study serum NfL levels were associated with neuropsychological test and brain atrophy scores [11].

In independent cohorts of nonagenarians (n = 180) and in centenarians (n = 135), higher plasma NfL levels were associated with lower survival probability [12]. More recently, a study conducted among 294 African Americans (baseline ages 49–65 y), found that after 14–15 years of follow up, baseline serum.

NfL levels were significantly higher in the decedent group (86.1 ± 65.7 pg/ml vs. 50.1 ± 28.0 pg/ml, *p* < 0.001) [13]. In multivariable-adjusted binomial logistic regression models, which included age, sex, education, baseline smoking status, BMI, and total comorbidities (0–11), serum NfL levels retained its predictive value for all-cause mortality, and sensitivity analyses included other covariates did not alter this key finding [13].

Collectively, these data suggest that blood-based NfL may be an indicator of neural deterioration that may hold predictive value for mortality but more studies are warranted to decipher the relationship of this biomarker to mortality in non-demented adults. Furthermore, given differences in associations of NfL with mortality in men and women, it is important to examine differences overall and by sex and account for cardiometabolic factors that may influence or mediate NfL levels [14, 15]. In fact, NfL has been linked to both the body mass index (BMI) [14, 16] and kidney disease [17–19] in previous studies. Studies show that NfL is associated with brain MRI diffusion-weighted metrics such as fractional anisotropy and mean diffusivity [18–22]. In turn, these metrics were previously shown to be linked with serum vitamin D3 status or 25-hydroxyvitamin D3 [25(OH)D3), serum folate and vitamin B-12, elevated blood pressure, various measures of insulin resistance and hyperglycemia including glycated hemoglobin (HbA1c), dyslipidemia and various co-morbidities [23–25]. In addition, given its strong association with mortality risk, self-rated health may act as a mediator between NfL and mortality.

The present study examines the association between serum NfL and all-cause mortality in a national sample of community-dwelling US adults aged 20–85 years at baseline and followed for up to 6 years until end of 2019. Associations were tested overall and by sex. As a secondary objective, the study also tests potential interactive and mediating effects of the body mass index (BMI), and other related measures of cardio-metabolic and general health and selected nutritional biomarkers on this relationship.

## Materials and methods

### Database

The NHANES consists of a series of surveys by the National Center for Health Statistics, a division of the Centers for Disease Control and Prevention (CDC), to evaluate the health and nutritional status of civilian, noninstitutionalized U.S. children and adults and to determine the burden of major diseases and their risk factors [26, 27]. In the NHANES, stratified multistage cluster sampling is used with oversampling [28] of specific groups. Demographic, socioeconomic, and nutritional data are collected through in-person interviews, physical examinations and laboratory tests [29]. Recent waves of NHANES data have over-sampled low-income persons, adolescents 12–19 years, individuals ≥ 60 years of age, African Americans, and Mexican Americans. Beginning in 2007–2008, NHANES oversampled all Hispanics, instead of only Mexican American, low-income persons, individuals ≥ 60 years of age, and African American individuals [28]. In addition, the “Asian” group was oversampled after 2011. The original study was approved by an Institutional Review Board with informed consent provided by all study participants.

### Study sample

**Fig. 1:**
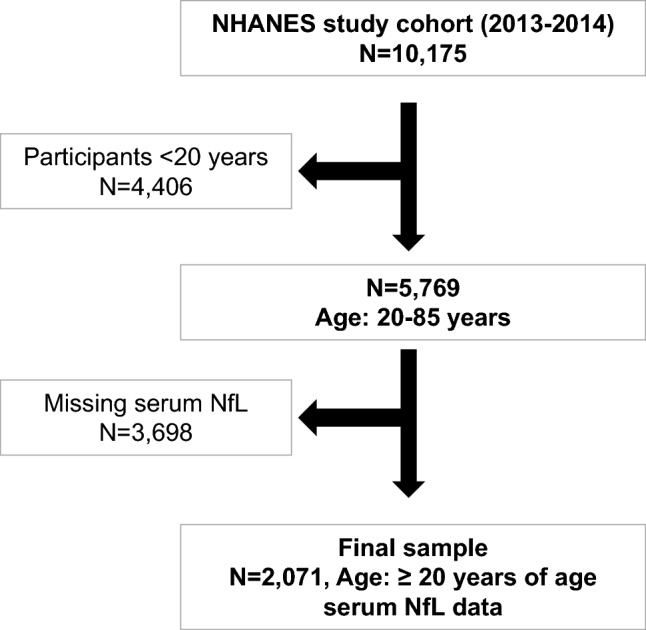
Participant flowchart: NHANES 2011–2014. *Abbreviations*: NfL = Neurofilament Light Chain; NHANES = National Health and Nutrition Surveys

The NHANES has been a continuous surveillance system, since 1999. For this study, the initial sample consisted of 10,175 NHANES participants from a single wave (2013–2014). Participants who were < 20 years of age (n = 4,406) were excluded, retaining 5,769 with age range of 20-85y at baseline examination. Of this subgroup, 3,698 were excluded for having missing data on serum NfL. Thus, the final sample consisted of 2,071 adults, ≥ 20 years of age, with complete data on serum NfL. No other exclusions were made for most analyses and all other covariates included in our analyses were imputed, using chained equations [30, 31] with details provided under “Statistical analysis” section (Fig. 1). On average, covariates aside from NfL were missing on 3–4% of the final sample of N = 2,071.

### Mortality linkage

The National Center for Health Statistics (NCHS) has linked data collected from several NCHS population surveys with death certificate records from the National Death Index (NDI). In compliance with requirements to protect the confidentiality of the NCHS survey participants, restricted-use versions of the linked mortality files are made available only through the NCHS Research Data Center (RDC). To complement the restricted-use files and increase data access, NCHS also developed public-use versions of the linked mortality files for the 1999–2014 National Health and Nutrition Examination Survey (NHANES) among others. The public-use linked mortality files include a limited set of variables for adult participants only. To reduce the participant disclosure risk, the public-use versions of the NCHS linked mortality files were subjected to data perturbation techniques. Synthetic data were substituted for follow-up time and underlying cause of death for select records. Information regarding vital status was not perturbed. The public-use linked mortality file provides mortality follow-up data from the date of survey participation through December 31, 2019. Detailed description of the linkage methodology and analytic guidelines can be found on the NCHS Data.

Linkage webpage: https://www.cdc.gov/nchs/data/datalinkage/LMF2015_Methodology_Analytic_Considerations.pdf

### Serum neurofilament light chain measurement

Eligible NHANES 2013–2014 participants who were aged 20–75 years consented to store their blood samples for future research and consisted of individuals who had stored surplus or pristine serum samples. Using a highly sensitive methods, NfL was measured by immunoassay using acridinium ester (AE) chemiluminescence and paramagnetic particles. This assay can be conducted on an existing, high-throughput, automated platform (Attelica). Strict quality assurance was applied and subsample weights were generated to analyze the data properly. The lower and upper limits of quantification were 3.9 pg/mL and 500 pg/mL, respectively. Other details are provided elsewhere (https://wwwn.cdc.gov/Nchs/Nhanes/2013-2014/SSSNFL_H.htm).

### Covariates

Socio-demographic characteristics were defined as follows: age (in y), sex (M/F), race (Non-Hispanic White, Non-Hispanic Black, Mexican American or other Hispanic, and Other race/ethnicities including Non-Hispanic Asian), level of education (< 9th grade or High school, 9–11th Grade, HS graduate, GED or equivalent, some college or Associate’s degree, ≥ College Graduate), marital status (married and/or living with partner vs. other) and poverty status as indicated by the poverty-income ratio [PIR] (< 100%, ≥ 100% to < 200% and ≥ 200%). Lifestyle characteristics were focused on smoking status (non-smoker vs. ex-smoker vs. current smoker), alcohol consumption ≥ 12 glasses (in past 12 months) [Y/N], ever use marijuana, cocaine, heroin or methamphetamine (Y/N). Physical activity in the past 30 days (moderate activity or vigorous activity, work or leisure; walk or bicycle) using a set of self-reported responses based on the short form of the International Physical Activity Questions [32] in terms of frequency (# of days) per week and number of minutes per day. Those were then combined to generate MET.min/week for each category of physical activity intensity. Finally, the MET.min/week values were added together. The NHANES 2013–14 included a dietary component consisting of two 24-h recalls administered by trained Mobile Examination Center (MEC) interviewers. The U.S. Department of Agriculture (USDA) by computerized Automated Multiple Pass Method was used to collect dietary intake data [33]. The first dietary recall interview was collected in-person in the MEC and the second interview was collected by telephone 3 to 10 days later. Nutrient intakes were estimated by linking dietary intake with corresponding USDA’s Food and Nutrient Database for Dietary Studies databases [34, 35]. The average daily nutrients intake from the two 24-h recalls was used in our current analysis. In our present study, the formula reported by Mellen et al. [36] was used to calculate the DASH diet score. This score is divided into nine target nutrients, specifically total fat, saturated fat, protein, fiber, cholesterol, calcium, magnesium, sodium and potassium. Micronutrient goals were expressed per 1000 kcal. The total DASH score was based on the sum of all nutrient targets met. If the participant achieved the DASH target for a nutrient, a value 1 was assigned. A value of 0.5 was given if the intermediate target was achieved, while a value of zero was assigned if neither target was met. Each of the nine components of the DASH total score were considered as separate exposures in our current study, in addition to the total score. In addition to dietary intakes, 3 nutritional biomarkers were included among potential confounding covariates, namely RBC folate, serum vitamin D3 [25(OH)D3] and serum vitamin B-12. Those are described in more detail in supplementary methods 1. Health characteristics were considered among potential mediators/moderators in the association between serum NfL and all-cause mortality. Those included body mass index (BMI) categories, self-rated health (SRH), a co-morbidity index and several measures of cardiometabolic health: systolic and diastolic blood pressure (mm Hg., average of 3 readings), glycated hemoglobin (HbA1c), total cholesterol and urinary albumin:creatinine ratio, ACR (Log_e_ transformed with outliers excluded), (supplementary methods 1). The co-morbidity index was a binary [Y/N] variable for any of several cardio-vascular and cancer morbidities, namely congestive heart failure, coronary heart disease, angina/angina pectoris, heart attack, stroke or cancer/malignancy. SRH was operationalized by one question: “Would you say your health in general is- excellent, very good, good, fair or poor?” and further dichotomized as “excellent/very good/good” as the referent category (“0”) vs. “fair/poor” coded as “1”.

### Statistical methods

We used Stata release 17 [37] to perform all descriptive and inferential analyses, accounting for sampling design complexity by including sampling weights, primary sampling units and strata in parts of the analyses. Aside from outcome and exposures, data was imputed using chained equations or MICE (5 imputations, 10 iterations) [30, 31], with most covariates having < 10% missing data compared to the final eligible sample (i.e. N = 2,071). MICE is a statistical technique that iteratively generates multiple imputations for each missing value in a dataset [30, 31]. This is accomplished by fitting predictive models to the observed data and assigning missing values progressively, one variable at a time, using information from other variables [30, 31]. A predictive model is created for each incomplete variable, using observed values of other variables to approximate the missing values [30, 31]. The imputation procedure is continued for a set number of iterations, typically until convergence is reached [30, 31]. At each iteration, missing values are updated using the most recent imputations, allowing imputation models to be refined [30, 31]. In our study, this is done for 10 iterations per imputation for a total of 5 imputations. Main Stata commands used included *mi impute*, *mi passive* and *mi estimate* in all the analyses. Study design complexity and survival time setting was specified using *mi stset* and *mi* *svyset* among others. Only potentially confounding and mediating/moderating covariates were imputed within the final selected sample with complete exposure and outcome data. The overall analytic sample was characterized at baseline using means and proportions. A series of bivariate and multivariable regression models were constructed to evaluate whether baseline characteristics varied according to sex, while accounting for sampling design complexity. To examine associations among plasma NfL exposures, we estimated a series of Cox proportional hazard regression models with sequential covariate adjustment. Time on study (months) was used as the initial underlying scale and was used to generate age at death accounting for initial age at baseline assessment in the NHANES. Sex-specific Kaplan–Meier survival curves were presented for binary NfL exposures (> vs. ≤ median) based on distributions in the final selected sample that has a cutoff of 2.51 for NfL on the Log_e_ transformed scale, while examining time on study as the analytic time variable. In Cox proportional hazards models, heterogeneity by sex of the association between NfL exposures and mortality was tested through addition of two-way interaction terms (NfL × sex) in separate models. The same models were also stratified by sex. Similarly, and as a secondary analysis, interaction by baseline age was tested for the main exposure, overall and within each gender group. The general modeling strategy consisted of a basic model, adjusted for age, sex, race and PIR (Model 1), to which other lifestyle and health-related covariates (listed in the Covariates section) were subsequently added (Model 2).

BMI, measures of cardio-metabolic health (SBP, DBP, total cholesterol, HbA1c and ACR), co-morbidity index, self-rated health and nutritional biomarkers (RBC folate, serum 25(OH)D3, and serum vitamin B-12) were separately assessed as mediating/interactive factors in the total effect of NfL exposures on all-cause mortality in the full Model 2. All other covariates in Model 2 as previously described were considered potential confounders. Continuous potential mediators were transformed into standardized z-scores, while indices were coded as 0 = no, 1 = yes/any, for ease of interpretation. Specifically, the overall effect of each main exposure on all-cause mortality (*Y*), in the presence of a mediator with which the exposure *a* may interact (*M*), was decomposed into four distinctive components, using the following general form of the model, accounting for potentially confounding covariates *c* [38]. Therefore, in all analyses, exposure levels were *a* = 1 and *a*’ = 0.

$$\begin{gathered} E\left[ {Y|\left( {a,m,c} \right)} \right] = \theta_{0} + \theta_{1} a + \theta_{2} m + \theta_{3} a*m + \theta_{c} c \hfill \\ E\left[ {M|\left( {a,c} \right)} \right] = \beta_{0} + \beta_{1} a + \beta_{c}^{T} c \hfill \\ \end{gathered}$$

(i) Neither mediation nor interaction or controlled direct effect (*CDE*): E[*CDE* | *c*] = *θ*_*1*_ (*a*–*a’*). This component is interpreted as the effect of the exposure on the outcome, not related to interaction or mediation [38].
(ii) Interaction alone (and not mediation) or interaction reference (*INT*_*ref*_): E[*INTref* | *c*] = θ_3_(β_0_ + β_1_a’ + β_c_^T^ *c*)(*a*–*a’*). This component is interpreted as the interaction effect of the exposure on the outcome in the presence of the mediator, when the presence of the outcome is not necessary for the presence of the mediator [38].
(iii) Both mediation and interaction or mediated interaction (*INTmed*): E[*INTmed* | *c*] = θ_3_ β_1_ (a–a’)(a–a’). This component is interpreted as the interaction effect of the exposure on the outcome in the presence of the mediator, when the presence of the outcome is necessary for the presence of the mediator [38].
(iv) Only mediation (but not interaction), or pure indirect effect (*PIE*): E(*PIE* | *c*] = (θ_2_ β_1_ + θ_3_ β_1_a’)(a-a’). This component is interpreted as the effect of the mediator on the outcome when the exposure is necessary for the presence of the mediator [38].

The total effect of the exposure on the outcome (*TE* = *CDE* + *INT*_*ref*_ + *INT*_*med*_ + *PIE*), is the summation of those four partitioned components that explain the total variance between the exposure and the outcome [38]. This four-way decomposition unifies methods that attribute effects to interactions and methods that examine mediation, and this method has recently been introduced in Stata, allowing to estimate four-way decomposition using parametric or semi-parametric regression models. Importantly, the *Med4way* command [39] [https://github.com/anddis/med4way] was used to test mediation and interaction of the total effects of NfL exposure on mortality with several mediators/effect modifiers, using Cox PH models for the outcome and linear or logistic regression models for each mediator/effect modifier. In this study, four-way decomposition was applied to the total sample. A logit link was specified for the mediating variable equation when mediators were binary. Total effects were interpreted as hazard ratios on the Log_e_ scale based on Cox proportional hazards models, per SD of exposures if the exposure was continuous and for “exposed” vs. “unexposed” if the exposure was binary. These total effects were then decomposed into four components. An effect size that would result in a hazard ratio > 1.5 was considered as moderate-to-strong. Unlike all other analyses, four-way decomposition models did not account for sampling design complexity.

In all models, we adjusted for sample selectivity due to missing exposure and outcome data, relative to the initially recruited sample, using a two-stage Heckman selection strategy [40]. Initially, we predicted an indicator of selection with socio-demographic factors, namely, age, race, sex and PIR using probit regression, which yielded an inverse mills ratio (IMR)—a function of the probability of being selected given those socio-demographic factors. Subsequently, we estimated our Cox proportional hazards regression models adjusted for the IMR in addition to afore-mentioned covariates [40, 41].

Two supplementary analyses were also carried out. In a first supplementary analysis, all variables of interest including potential mediators/moderators and exogenous variables were compared across exposure levels (above vs. below median) using bivariate linear and multinomial logistic models on multiple imputed data. The second supplementary analysis specifically examined the association between each potential moderator with all-cause mortality (multivariable-adjusted Cox PH model with only main effect of the potential moderator: Model A); tested interactions between Log_e_ transformed and z-scored NfL and those variables of interest in relation to all-cause mortality (multivariable-adjusted Cox PH model with 2-way interaction between NfL and each potential moderator: Model B); and associations of potential mediators/moderators with NfL while adjusting for exogenous covariates (multiple linear regression models, multiple-imputed data: Model C). In Model B, interaction was tested on the multiplicative scale. Conversion to the additive scale can be implied with equations shown below for the Cox proportional hazards model and the relative excess risk due to interaction (RERI), when both exposures and moderators are binary [42–44]. This sub-analysis was carried out with exposure (LnNfL, z-scored) and continuous moderators transformed into below and above median binary variables, as well as the remaining binary potential moderators. RERI_HR_ formula is shown below with IR being the estimated incidence rates within each group, conditioning simultaneously on exposure and potential moderator [42–44]. For each combination of exposure and potential moderator level, an excess relative risk was computed as shown below [42–44]. The attributable proportion (AP) statistic is the proportion of risk for the doubly exposed (**+ + **or 11) interaction that is due to the risk that is above additive. The synergy index (SI) statistic is the excess risk expressed as a ratio rather than a difference, with a value > 1 indicating synergy or super-additive effects [42–44]. Exogenous covariates included age, sex, race/ethnicity, PIR, education, smoking, ever drug use, alcohol use, DASH, total caloric intake, physical activity, household size and marital status. All Stata codes used and secondary Output in this study will be provided under the following repository: baydounm/NHANES_NfL_mortality (github.com).

$$\lambda (t;X,M,C) = \lambda_{0} (t)e^{{\beta_{1} X + \beta_{2} M + \beta_{3} XM + \sum\limits_{k = 1}^{n} {\gamma_{k} C_{k} } }}$$

$$\begin{gathered} RERI_{HR} = e^{{\beta_{1} + \beta_{2} + \beta_{3} }} - e^{{\beta_{1} }} - e^{{\beta_{2} }} + 1 = HR(1,1;c) - HR(1,0;c) - HR(0,1;c) + 1 \hfill \\ = \frac{{IR_{11} - IR_{10} - IR_{01} + IR_{00} }}{{IR_{00} }} \hfill \\ \end{gathered}$$

$$ERR = IR_{am} /IR_{00} - {\text{1, where}}\;a\;{\text{and}}\;m\;{\text{are exposure and potential moderator's values as }} 0 \, \left( - \right){\text{ or 1 }}\left( + \right).$$

$$\begin{gathered} AP = RERI_{HR} /IR_{11} \hfill \\ SI = \left( {IR_{11} - 1} \right)/\left[ {\left( {IR_{10} - 1} \right) + \left( {IR_{01} - 1} \right)} \right] \hfill \\ \end{gathered}$$

## Results

Our analytic sample consisted of 2,071 participants aged 20-85y with complete data on serum NfL. Mean age at exam in the total sample was 45.1 y, and 84 deaths occurred over a mean follow-up time of 70.6 months (SD 10.4, IQR 65–78 months). Table 1 shows that on average, Log_e_ transformed serum NfL was 2.54 and was significantly higher among men compared to women (2.59 vs. 2.48, *P* = 0.001). Men were more likely to have < HS education compared to women (5.2% vs. 3.4%, *P* = 0.003), while women attained some college education at a greater proportion than men (36.2% vs. 30.7%). Women were more likely than men to be unmarried/unpartnered (37.7% vs. 32.3%, *P* = 0.008), and to live below the poverty line (PIR < 100%: 19.8% vs. 16.5%, *P* = 0.042). There were greater odds of being an ex-smoker (28.4% vs. 16.6%, *P* < 0.001) or current smoker (21.9% vs. 20.7%, *P* = 0.017) among men compared to women, as opposed to being a never smoker (49.7% vs. 62.7%). Similarly, men were more likely than women to be alcohol consumers over the past 12 months and drug ever users (*P* < 0.001). Nevertheless, physical activity measured using metabolic equivalents × minutes/week (Met.min.wk^−1^), was greater among men compared to women (2,652 vs. 1,496, *P* = 0.005). Sex differences in dietary factors, nutritional biomarkers, cardiometabolic factors and measures of general health status were also detected. Specifically, while caloric intake was greater among men, DASH total score, reflecting diet quality, was higher among women (2.26 vs. 2.02, *P* = 0.003). Women had higher mean BMI, total cholesterol, urinary ACR, glycated hemoglobin, serum 25(OH)D3 and B-12, as well as higher RBC folate compared with men, while the reverse was true for SBP and DBP. No sex differences were detected in self-rated health and co-morbidity measures .

**Table 1:** Study sample characteristics, overall and by sex: NHANES 2013–2014

	Overall		Men		Women		P_sex_
	N = 2,071		N = 990		N = 1,081		
	Mean/%	(SE)	Mean/%	(SE)	Mean/%	(SE)	
Exposures and outcomes							
NfL, Log_e_ transformed Mean (SEM)	2.54	(0.03)	2.59	(0.04)	2.48	(0.04)	0.001
NfL, above median, %	48.2	(1.7)	50.8	(2.1)	45.7	(2.0)	0.048
Died, %	3.5	(0.5)	3.5	(0.8)	3.5	(0.5)	0.91
Covariates							
Age (years):	45.1	(0.5)	44.7	(0.5)	45.4	(0.59)	0.28
Mean (SEM)							
% Women	51.3	(0.8)	–		–		
Race/Ethnicity							Ref
Non-Hispanic White	64.9	(3.6)	65.8	(3.9)	64.2	(3.6)	0.21
Non-Hispanic Black	12.0	(1.6)	11.2	(1.5)	12.8	(1.8)	0.69
Mexican–American and other Hispanic	15.3	(2.7)	15.8	(3.0)	14.9	(2.5)	0.33
Other	7.7	(1.0)	7.2	(1.2)	8.1	(1.0)	
Household size	3.21	(0.07)	3.26	(0.09)	3.20	(0.10)	0.19
Education							
< Less Than 9th Grade	4.3	(0.7)	5.2	(0.9)	3.4	(0.5)	0.003
9–11th Grade	11.4	(1.3)	11.8	(1.7)	11.1	(1.2)	0.14
High School Grad/GED or Equivalent	20.1	(1.5)	21.7	(1.7)	18.6	(1.9)	0.018
Some College or AA Degree	33.5	(1.4)	30.7	(1.6)	36.2	(1.7)	Ref
College Graduate or Above	30.7	(2.2)	30.7	(2.4)	30.7	(2.4)	0.085
Marital Status							
Married/Living with Partner	64.9	(1.7)	67.7	(1.9)	62.3	(1.8)	Ref
Other	35.1	(1.7)	32.3	(1.9)	37.7	(1.8)	0.008
Poverty-Income Ratio							
< 100%	18.1	(2.3)	16.5	(2.5)	19.8	(2.3)	0.042
100– < 200%	19.6	(1.3)	19.1	(1.7)	20.0	(1.3)	0.25
≥ 200%	62.3	(3.4)	64.5	(3.8)	60.2	(3.3)	Ref
Smoking Status							
Never Smoker	56.4	(2.3)	49.7	(2.6)	62.7	(2.4)	Ref
Ex-Smoker	22.4	(1.6)	28.4	(2.0)	16.6	(1.4)	< 0.001
Current Smoker	21.3	(2.1)	21.9	(2.1)	20.7	(2.5)	0.017
Alcohol Consumption (≥ 12 glasses in past 12 months):							
Yes	77.6	(2.5)	85.9	(2.2)	69.6	(3.2)	Ref
No	22.4	(2.5)	14.1	(2.2)	30.4	(3.2)	< 0.001
Drug ever use, %	45.5	(2.1)	52.7	(2.6)	38.8	(2.2)	< 0.001
Physical Activity, Met.min.wk^−1^	2,059	(208)	2,652	(306)	1,496	(231)	0.005
Body Mass Index *(kg/m*^*2*^*)*:							
Mean (SEM)	29.4	(0.3)	28.7	(0.3)	30.0	(0.3)	0.002
Systolic Blood Pressure *(mm Hg)*:							
Mean (SEM)	119.9	(0.6)	121.5	(0.7)	118.3	(0.7)	0.003
Diastolic Blood Pressure (*mm Hg*): Mean (SEM)	69.2	(0.5)	70.3	(0.7)	68.2	(0.4)	0.007
Total cholesterol, mmol/L	4.89	(0.04)	4.83	(0.04)	4.95	(0.05)	0.029
Mean (SEM)							
Glycated Hemoglobin, %	5.60	(0.02)	5.63	(0.03)	5.58	(0.03)	0.26
Urinary albumin:creatinine ratio, Log_e_ transformed	2.13	(0.03)	1.96	(0.03)	2.31	(0.04)	< 0.001
Serum vitamin D3, 25(OH)D3, nmol/L	64.3	(1.3)	61.2	(1.3)	67.2	(1.8)	0.003
RBC folate, nmol/L	1,242	(26.1)	1,214	(24.9)	1,269	(31.1)	0.019
Serum vitamin B-12, pmol/L	602.1	(23.2)	548.0	(13.8)	653.3	(39.8)	0.016
Self-rated health							
Excellent/Very Good/Good	81.7	(1.8)	83.1	(2.1)	80.0	(1.8)	Ref
Fair/Poor	18.3	(1.8)	16.9	(2.1)	19.5	(1.8)	0.12
Co-morbidity, %	14.6	(0.9)	15.6	(1.6)	13.6	(1.0)	0.34
Energy intake, kcal/d	2,117	(23.3)	2,431	(35.7)	1,820	(25.3)	< 0.001
DASH diet total score							
Mean (SEM)	2.15	(0.05)	2.02	(0.06)	2.26	(0.06)	0.003

*25(OH)D3* 25-hydroxy vitamin D3, *DASH* Dietary Approaches to Stop Hypertension, *nc* not computed, *NfL* Neurofilament Light Chain, *NHANES* National Health and Nutrition Examination Surveys, *SEM* Standard Error of the Mean. *N* = 989 among men and *N* = 1,081 among women

**Fig. 2:**
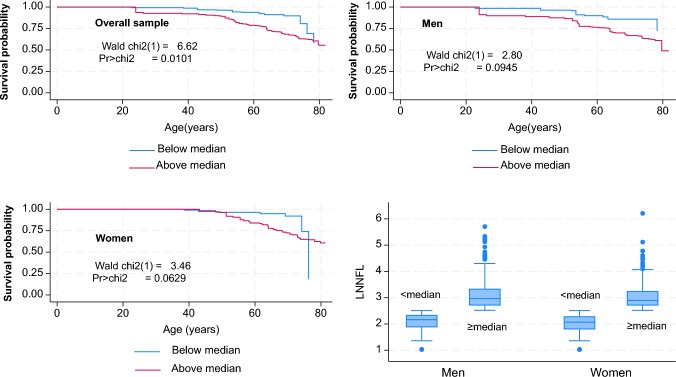
Survival probability above and below median NfL plasma levels (Log_e_ transformed), overall and by sex: Kaplan–Meier survival curves: NHANES 2011–2014. *Abbreviations*: Neurofilament Light Chain; NHANES = National Health and Nutrition Surveys

Our key findings are presented in Fig. 2 and Tables 2 and 3. Figure 2 shows a set of Kaplan–Meier survival curves across two groups of Log_e_ NfL levels (above vs. below median). Results indicated that mortality risk was greater in the above median group compared to the group below median NfL, particularly in the overall sample (*P* = 0.010), with trends observed within each sex group (*P* < 0.10). When looking at Log_e_ NfL as a continuum as shown in Table 2, each SD was associated with a significantly increased mortality risk (HR 1.88, 95% CI 1.60–2.20, *P* < 0.001) in the reduced model adjusted for age, sex, race, and poverty income ratio; a finding that was attenuated with addition of lifestyle and health-related factors (which included among other HbA1c and ACR); (HR 1.67, 95% CI 1.41–1.98, *P* < 0.001). This attenuation was mainly observed in men (HR 1.90 (*P* = 0.001) in Model 1 vs. 1.70 (*P* = 0.004) in Model 2), while the reverse pattern was observed among women (HR 1.65 (*P* = 0.002) in Model 1 vs. 1.83 in Model 2 (*P* = 0.021)). Little evidence of heterogeneity was found by age, especially after further adjustment for lifestyle and health-related covariates.

**Table 2:** Plasma NfL (Log_e_ transformed, z-scored) and its relation to all-cause mortality, overall, by sex and with interaction by age, NHANES 2013–2014: Cox PH hazards models

	Log_e_(HR)	(SE)	*P*	HR	95% LCL	95% UCL
*Overall, n = 2,070*						
Model 1						
zLnNfL	+ 0.632	(0.082)	< 0.001	1.88	1.60	2.20
Model 2						
zLnNfL	+ 0.513	(0.088)	< 0.001	1.67	1.41	1.98
Model 3						
zLnNfL	+ 0.654	(0.084)	< 0.001	–	–	–
Age	− 0.098	(0.079)	0.24	–	–	–
zLnNfL × Age	− 0.002	(0.004)	0.52	–	–	–
Model 4						
zLnNfL	+ 0.565	(0.096)	< 0.001	–	–	–
Age	− 0.126	(0.069)	0.089	–	–	–
zLnNfL × Age	− 0.006	(0.004)	0.16	–	–	–
*Men, n = 989*						
Model 1						
zLnNfL	+ 0.640	(0.154)	0.001	1.90	1.40	2.56
Model 2						
zLnNfL	+ 0.534	(0.152)	0.004	1.71	1.27	2.30
Model 3						
zLnNfL	+ 0.625	(0.140)	0.001	–	–	–
Age	− 0.098	(0.099)	0.38	–	–	–
zLnNfL × Age	+ 0.006	(0.003)	0.070	–	–	–
Model 4						
zLnNfL	+ 0.534	(0.155)	0.004	–	–	–
Age	− 0.244	(0.123)	0.070	–	–	–
zLnNfL × Age	− 0.004	(0.008)	0.63	–	–	–
*Women, n = 1,081*						
Model 1						
zLnNfL	+ 0.500	(0.132)	0.002	1.65	1.27	2.14
Model 2						
zLnNfL	+ 0.604	(0.230)	0.021	1.83	1.17	2.87
Model 3						
zLnNfL	+ 0.718	(0.263)	0.017	–	–	–
Age	− 0.097	(0.101)	0.35	–	–	–
zLnNfL × Age	− 0.016	(0.019)	0.43	–	–	–
Model 4						
zLnNfL	+ 0.542	(0.259)	0.058	–	–	–
Age	− 0.131	(0.090)	0.18	–	–	–
zLnNfL × Age	+ 0.007	(0.027)	0.79	–	–	–

*NfL* Neurofilament Light Chain, *NHANES* National Health and Nutrition Examination Surveys, S*EM* Standard Error of the Mean. *N* = 989 among men and *N* = 1,081 among women; zLnNfL = Log_e_ transformed NfL, z-scored

Models 1 and 3 adjusted for age, sex, race and poverty income ratio categories, and the inverse mills ratio. Model 2 and 4 further adjusted for other lifestyle and health-related factors listed under “Covariates” section. Models 3 and 4 included an age by exposure 2-way interaction term. Age in these models is centered at 47 y

**Table 3:** Plasma NfL (Log_e_ transformed, z-scored) and its relation to all-cause mortality, overall and by sex: mediating and interactive effects of cardiometabolic and general health factors and of nutritional biomarkers using four-way decomposition: NHANES 2013–2014

	Overall (N = 2,071)	
	β ± SE	*P*
*X = NFL; M = Body Mass Index*		
Total effect	**+ 0.612 ± 0.194**	**0.002**
CDE	**+ 0.628 ± 0.191**	**0.001**
INTref	− 0.018 ± 0.026	0.49
INTmed	− 0.015 ± 0.017	0.39
PIE	+ 0.016 ± 0.012	0.18
*X = NFL1; M = Systolic Blood Pressure*		
Total effect	**+ 0.618 ± 0.183**	**0.001**
CDE	**+ 0.664 ± 0.191**	**0.001**
INTref	− 0.001 ± 0.014	0.91
INTmed	− 0.010 ± 0.012	0.41
PIE	− 0.035 ± 0.033	0.30
*X = NFL; M = Diastolic Blood Pressure*		
Total effect	**+ 0.672 ± 0.197**	**0.001**
CDE	**+ 0.672 ± 0.197**	**0.001**
INTref	+ 0.007 ± 0.020	0.71
INTmed	− 0.007 ± 0.008	0.34
PIE	− 0.001 ± 0.011	0.95
*X = NFL; M = Glycated hemoglobin (HbA1c)*		
Total effect	**+ 0.512 ± 0.220**	**0.020**
CDE	**+ 0.523 ± 0.199**	**0.008**
INTref	− 0.011 ± 0.022	0.59
INTmed	**+ 0.032 ± 0.014**	**0.027**
PIE	− 0.033 ± 0.031	0.28
*X = NFL; M = Total serum cholesterol*		
Total effect	**+ 0.697 ± 0.213**	**0.001**
CDE	**+ 0.710 ± 0.219**	**0.001**
INTref	− 0.007 ± 0.029	0.80
INTmed	− 0.0009 ± 0.0026	0.72
PIE	− 0.0051 ± 0.0082	0.54
*X = NFL; M = Albumin:Creatinine ratio*		
Total effect	**+ 0.611 ± 0.212**	**0.004**
CDE	**+ 0.532 ± 0.209**	**0.011**
INTref	**+ *0.052* ± *0.031***	***0.094***
INTmed	**+ *0.017* ± *0.010***	***0.084***
PIE	+ 0.010 ± 0.013	0.44
*X = NFL; M = Serum Vitamin D*		
Total effect	**+ 0.611 ± 0.196**	**0.002**
CDE	**+ 0.635 ± 0.191**	**0.001**
INTref	0.036 ± 0.037	0.32
INTmed	− 0.025 ± 0.016	0.11
PIE	− **0.036 ± 0.017**	**0.031**
*X = NFL; M = Red blood cell folate*		
Total effect	**+ 0.746 ± 0.202**	**< 0.001**
CDE	**+ 0.756 ± 0.203**	**< 0.001**
INTref	− 0.0201 ± 0.0150	0.18
INTmed	− 0.0050 ± 0.0069	0.46
PIE	**+ *0.0153* ± *0.008***	***0.065***
*X = NFL; M = Serum Vitamin B-12*		
Total effect	**+ 0.647 ± 0.208**	**0.002**
CDE	**+ 0.614 ± 0.204**	**0.003**
INTref	**+ *0.024* ± *0.013***	***0.057***
INTmed	+ 0.006 ± 0.006	0.30
PIE	+ 0.002 ± 0.003	0.52
*X = NFL; M = Co-morbid conditions*		
Total effect	**+ 0.822 ± 0.235**	**< 0.001**
CDE	**+ 0.893 ± 0.239**	**< 0.001**
INTref	− ***0.074* ± *0.038***	***0.054***
INTmed	− ***0.022* ± *0.013***	***0.086***
PIE	+ 0.026 ± 0.021	0.21
*X = NFL; M = Self-Rated Health*		
Total effect	**+ 0.691 ± 0.217**	**0.001**
CDE	**+ 0.577 ± 0.222**	**0.010**
INTref	+ 0.082 ± 0.061	0.18
INTmed	+ 0.014 ± 0.012	0.23
PIE	+ 0.018 ± 0.019	0.33

*CDE* Controlled Direct Effect, *INTmed* Interaction, mediated, *INTref* Interaction, Reference, *M* Mediators/Effect Modifier, *NfL* Plasma Neurofilament Light Chain, Log_e_ transformed, *PIE* Pure Indirect Effect, *X* Exposure

^a^See methods and Table 1 for definition of the NfL exposure. All exposures (X) and potential mediators/effect modifier (M) were z-scored for ease of interpretation, with the exception of binary M (coded as 0/1), namely co-morbidity and self-rated health. Control variables were set at their means

^b^Cox models for which four-way decomposition was conducted is equivalent to Model 2, Table 2, for continuous exposures, to which M was added and considered as a potential mediator/effect modifier. Control variables included age at, sex, race, poverty status, all remaining lifestyle and health-related factors and the inverse mills ratio

^c^Total effects are beta = Log_e_(HR) ± SE with associated *p*-values from Cox PH hazards models associated with each NfL exposure of interest. Hazard Ratios (HR) point estimates exponent of beta. 95% CI for HR can be calculated as follows: Lower confidence limit, LCL: exp[Log_e_HR-1.96SE(Log_e_HR)], upper confidence limit, UCL: exp[Log_e_HR + 1.96SE(Log_e_HR)]. Bolded values are for p<0.05; Bolded and italicized values are for P<0.10

Table 3 shows main findings derived from four-way decomposition models, which decomposed each total effect (TE) per mediator/moderator of the specified NfL exposure on all-cause mortality into components attributed to mediation alone (Pure Indirect Effect [PIE]), interaction alone (Reference Interaction [INT_ref_]), to both mediation and interaction (Mediated Interaction [INT_med_]), and neither mediation nor interaction (Controlled Direct Effect [CDE]). All total effects observed in the overall sample were statistically significant at a type I error of 0.05, and the direct effect was also significant and in the same direction as the total effect for all intermediate variables. For some of those intermediate variables, some indication of mediation and/or moderation was observed. Most notably, the IM component was statistically significant for HbA1c (INT_med_ =  + 0.032, *P* = 0.027 with a total effect of + 0.512 or a HR = 1.66, *P* = 0.020), indicating that 7% of this total effect of NfL on all-cause mortality, adjusting for the remaining covariates, is due to mediated interaction by HbA1c. Another notable finding is the pure mediation detected for 25(OH)D3 (PIE = − 0.036, *P* = 0.031; TE =  + 0.611; *P* = 0.002). This suggests that 25(OH)D3 may be positively associated with NfL in this overall sample, while being inversely related to the all-cause mortality outcome or vice versa. Our additional analysis confirmed the first pattern of association (Supplementary Table 1). Other findings indicated a trend towards pure mediation (RBC folate), and reference and mediated interaction (ACR and co-morbidity), (Table 3).

Supplementary Table 1 presents study sample characteristics according to NfL levels (above vs. below median). Elevated NfL was associated with older age, a higher proportion of NH White adults, smaller mean household sizes, greater proportion of current smokers, higher means of systolic blood pressure, glycated hemoglobin, urinary ACR, total cholesterol, serum vitamin D3, poorer general health and presence of co-morbidities.

Supplementary Table 2A shows additional findings for associations between potential mediators/moderators, all-cause mortality and the main exposure, Log_e_ transformed NfL. All key continuous variables (i.e. exposures and potential mediators/moderators) were *z*-scored. Models were fully adjusted for socio-demographic, lifestyle and health-related factors. Our findings suggested that there was a synergistic interaction between HbA1c and NfL exposure and that HbA1c was directly associated with both NfL and all-cause mortality. In contrast, ACR was linked to both NfL and all-cause mortality, though NfL was the only predictor for mortality in a model that included the 2-way interaction term, between NfL and ACR along with the main effects. Vitamin D status was an independent predictor of reduced mortality from NfL, with no interaction detected between the two variables, confirming our previous finding of pure though inconsistent mediation. Similarly, NfL’s association with all-cause mortality was stronger among individuals without cardiovascular or cancer-related co-morbidities and is an independent predictor of mortality from self-rated health. Finally, there was synergism detected between NfL and vitamin B-12 in relation to all-cause mortality, with B-12 positively associated with all-cause mortality, but not with NfL.

Examining these relationships on an additive scale with binary exposures and potential moderators (Supplementary Table 2B), the AP index was statistically significant indicative of a relatively higher proportion due to synergy for LnACR (above vs. below median) in its interaction with LnNfL(above vs. below median) in relation to all-cause mortality (e.g. first imputation: LnNfL(below median, 0), LnACR(above median, 1): excess relative risk (ERR) = (IR_01_/IR_00_)-1 =  + 0.085 ± 0.65, *p* = 0.89; LnNfL(above median, 1), LnACR(below median, 0): ERR = (IR_10_/IR_00_)-1 = 0.207 ± 0.654, *p* = 0.73; LnNfL(above median, 1), LnACR(above median, 1): ERR = (IR_11_/IR_00_)-1 =  + 2.45 ± 1.82, *p* = 0.019; RERI: 2.16 ± 1.16, *p* = 0.064; AP: 0.625 ± 0.213, *p* = 0.003; SI: 8.38). The remaining four imputations gave similar results and the full Output is provided on github: baydounm/NHANES_NfL_mortality (github.com).

## Discussion

We investigated the relationship between serum NfL and all-cause mortality in a national sample of US adults aged 20-85y at baseline and followed for up to 6 years until the end of 2019. Furthermore, we tested these associations both overall and stratified by sex. Moreover, our study is also among the first to test potential interactive and mediating effects of BMI, other measures of cardio-metabolic risk, general health status and selected nutritional biomarkers on the main NfL-mortality relationship. Results indicated that mortality risk was greater in the above median NfL group compared to the group below median NfL, particularly in the overall sample (*P* = 0.010), with trends observed within each gender group (*P* < 0.10). When looking at Log_e_ NfL as a continuum, each SD was associated with a markedly increased mortality risk (HR 1.88, 95% CI 1.60–2.20, *P* < 0.001) in the reduced model adjusted for age, sex, race, and poverty income ratio; a finding that was attenuated with addition of lifestyle and health-related factors (HR 1.67, 95% CI 1.41–1.98, *P* < 0.001). Four-way decomposition indicated that there was, among others, mediated interaction between NfL and HbA1c and pure mediation with 25(OH)D3 in predicting all-cause mortality, in models adjusted for all other covariates. The former suggests that there was significant interaction effect of the higher NfL exposure on mortality risk with higher HbA1c, when increased mortality risk is necessary for elevated HbA1c. The latter suggests an inconsistent type of mediation in which TE and CDE were positive, while PIE was negative [39, 45, 46]. Specifically, NfL was positively associated with 25(OH)D3 which was then inversely associated with mortality risk. More generally, the significant PIE suggests that the effect of the mediator on the outcome when the exposure is necessary for the presence of the mediator is an inverse one, suggesting that NfL may be potentially in part protective against mortality risk through a mechanism that elevates 25(OH)D3. Furthermore, ACR interacted synergistically with NfL in relation to mortality risk both on the additive and multiplicative scales.

Emerging evidence indicates that elevated plasma NfL levels are associated with a multitude of neurological diseases including sporadic and familial AD [3, 5, 6], frontotemporal degeneration [47], multiple sclerosis [8], traumatic brain injury [7], Parkinson’s disease [2] and other neurological disorders [9]. Importantly, higher blood NfL levels precede the onset of clinical symptoms of AD [48, 49]. Plasma NfL levels may also have clinical value in middle-aged dementia-free adults. For example, in White adults and in those > 50 years, plasma NfL was associated with a faster decline on normalized mental status scores [41]. However, data from the Multidomain Alzheimer’s Preventive Trial (MAPT) indicated that plasma NfL was associated with cognitive scores and executive function only in older adults (median age 75 years) with MCI but not in individuals without cognitive impairment [50]. Therefore, blood NfL levels are associated with neurological diseases, but we are only beginning to understand the relationship between blood NfL and cognition in population-based studies and when and if NfL can have prognostic value in this context.

Recent attention has focused on characterizing blood NfL levels not only in neurological diseases and disorders but also with the associated adverse health outcomes. For example, there have been several reports that have associated plasma NfL levels with mortality due to stroke [51, 52], sporadic Creutzfeldt-Jakob disease [53], and spontaneous subarachnoid and intracerebral hemorrhages [54, 55]. In these studies, the follow-up periods were ~ 30 days with the exception of the Creutzfeldt-Jakob disease study which had a 14.8-month follow-up period. Thus, in these cases of acute neuroaxonal damage higher blood NfL levels were associated with short-term mortality. There have been a few recent reports that have examined whether blood NfL levels could predict all-cause mortality in population-based studies. Three recent studies found that plasma NfL was associated with all-cause mortality in centenarians and nonagenarians [12], among older (> 65 years) adults [11] and among African American middle-aged adults followed for over a decade [13]. In the MEMO study of elderly adults, there were no sex differences reported [11]. This is in contrast to a recent study of diverse middle-aged (mean age around 48 years) African American and White adults [10]. In this study, plasma NfL was associated with mortality only in women [10]. Here, we have added to these current studies by examining serum NfL in a diverse, representative sample of the US that ranged in age from 20 to 85 years. Individuals with levels of NfL in the above median group have a greater mortality risk compared to the group below median NfL. These findings are for the overall sample and only a trend when examined within each sex group. In terms of exploring possible associations through potentially mediating and/or moderating factors, in the HANDLS cohort, there was a possible antagonistic interaction between hsCRP and NfL, indicating that NfL is a better prognostic indicator at normal hsCRP values [10]. Moreover, there was some evidence of synergistic interaction between HbA1c and annualized change in plasma NfL (δNfL) in determining mortality risk, overall [10]. This latter finding is in line our present study, even though we only had data on a single point NfL exposure. Thus, our study further elucidates the relationship between serum NfL and all-cause mortality in the general population. Poor glycemic control and not the duration of diabetes was also found in a previous study to be independently associated with sNfL. Specifically, HbA1c levels were positively correlated to sNfL levels [β(SE)_adj_ = 1.85 (0.64), *p* < 0.01] [56]. This finding was corroborated by other studies among urban adults and in a comparable national sample [57, 58]. Our novel finding of pure inconsistent mediation with 25(OH)D3 is not supported by previous studies indicating that vitamin D supplementation had no detectable longitudinal effect on serum NfL in both animal and human studies [59, 60].

Our current study has several strengths, including the use of data from a large nationally representative sample of US adults with a wide age range (20–85 years) and a multi-racial/ethnic composition. In addition, this is among few studies to examine the association between serum NfL and mortality among community-dwelling, generally healthy US adults. The continuous NDI linkage to mortality data from baseline till end of 2019 allowed for enough statistical power to test our hypotheses. We used a number of advanced statistical techniques to conduct our analyses, including Cox proportional hazards models accounting for sampling design complexity and adjusting for potentially confounding covariates in multiple imputed data, 2-stage Heckman selection models to adjust for selection bias, and four-way decomposition models to tease out mediation from moderation.

Study findings should be interpreted in light of several limitations. First, even though cohort study designs are considered as the gold standard in epidemiological literature, cause-and-effect relationships cannot be clearly established in the context of observational studies. Second, sample sizes were likely too small to be able to detect interactions among variables of interest, including biomarkers and NfL in relation to all-cause mortality, and especially within sex groups. Thus, four-way decomposition models could not be constructed for men and women, separately, and its statistical power may be limited given the small number of deaths that occurred within the short period of time. The same limitation applied to interactions on the additive scale with binary exposures and potential moderators. Moreover, complete-subject analyses which depend on availability of data on key variables may have resulted in selection bias. Third, the role of chance cannot be ruled out given the large number of statistical tests being performed and thus our statistically significant findings should be interpreted in light of the multiplicity of statistical testing. Fourth, although many covariates were included in the multivariable models, residual confounding cannot be ruled out as an explanation for observed relationships. For example, total caloric intake was assessed using 24-h recall which may not be adequate for the determination of usual food consumption. Fifth, changes in NfL over time may be more important than NfL at a specific point in time. These data were not available in this wave of NHANES. Sixth, ultrasensitive protein assays have enabled accurate measurement of neuronal damage markers like NFL in blood [61]. Despite the inability to distinguish central from peripheral nerve degeneration, these biomarkers are effective in identifying patients with peripheral neuropathy and disease activity in rapidly progressive peripheral neuropathy [61]. However, their use in slowly progressive diseases without significant axonal loss remains uncertain [61]. Finally, four-way decomposition models rely on a strong set of assumptions, specifically that of no unmeasured confounding, which cannot be explicitly ruled out or tested empirically [62].

In conclusion, we report that serum NfL levels measured at the baseline MEC exam can predict all-cause mortality among both men and women. These findings merit further exploration in other large samples of adults, which will add to its usefulness as a potential prognostic marker at varying degrees of cardio-metabolic risk, particularly in terms of HbA1c levels. Our finding of pure inconsistent mediation through 25(OH)D3 merits further examination both in terms of replication and explanation through mechanistic studies. The same applies to the apparent synergism between NfL and urinary ACR in relation to mortality risk. It remains crucial to identify biomarkers for all-cause mortality, given the alarming increase in its rate among younger and middle-aged adults [63]. While, we did not observe any sex-specific findings, future studies should continue to examine heterogeneity in the NfL-mortality association across sex groups, given the sex difference in both NfL levels with age, and mortality. Together, this accumulating evidence suggests that serum levels of NfL are not only markers of neuropathology but can also predict mortality risk and thus may be useful for interventions aimed at elongating lifespan.

### Supplementary Information

Below is the link to the electronic supplementary material.

Supplementary file1 (DOCX 60 KB)

## Data Availability

The current analysis is based on public use data, available at: https://www.cdc.gov/nchs/nhanes/index.htm. Parts of the statistical code and/or Output can be requested from the corresponding author.

## Abbreviations

25(OH)D3: 25-Hydroxy vitamin D3
AD: Alzheimer's disease
AE: Acridinium-ester
BMI: Body mass index
CDC: Centers for Disease Control and Prevention
CDE: Controlled direct effect
CI: Confidence interval
CSF: Cerebrospinal fluid
CV: Coefficient of variation
DBP: Diastolic blood pressure
HR: Hazard ratio
HS: High school
IMR: Inverse mills ratio
INTmed: Mediated interaction
INTref: Interaction reference
IRB: Institutional Review Board
LLOQ: Lower limit of quantification
MAPT: Multidomain Alzheimer’s Preventive Trial
MEC: Mobile examination center
MEMO: Memory and Morbidity in Augsburg Elderly
MS: Multiple sclerosis
NCHS: The National Center for Health Statistics
NDI: National Death Index
NfL: Plasma neurofilament light
NHANES: National Health and Nutrition Examination Survey
OLS: Ordinary least squares
P: P-value
PIE: Pure Indirect effect
PMP: Paramagnetic particles
QC: Quality control
RDC: Research Data Center
SBP: Systolic blood pressure
SD: Standard deviation
SE: Standard error
ULOQ: Upper limit of quantification
US: United States
USDA: U.S. Department of Agriculture

## References

[1] Raket LL, Kuhnel L, Schmidt E, Blennow K, Zetterberg H, Mattsson-Carlgren N. Utility of plasma neurofilament light and total tau for clinical trials in Alzheimer’s disease. Alzheimers Dement (Amst). 2020;12(1):e12099. 10.1002/dad2.12099. 32995466 10.1002/dad2.12099 PMC7507310

[2] Hansson O, Janelidze S, Hall S, et al. Blood-based NfL: a biomarker for differential diagnosis of parkinsonian disorder. Neurology. 2017;88(10):930–7. 10.1212/WNL.0000000000003680. 28179466 10.1212/WNL.0000000000003680 PMC5333515

[3] Preische O, Schultz SA, Apel A, et al. Serum neurofilament dynamics predicts neurodegeneration and clinical progression in presymptomatic Alzheimer’s disease. Nat Med. 2019;25(2):277–83. 10.1038/s41591-018-0304-3. 30664784 10.1038/s41591-018-0304-3 PMC6367005

[4] de Wolf F, Ghanbari M, Licher S, et al. Plasma tau, neurofilament light chain and amyloid-beta levels and risk of dementia; a population-based cohort study. Brain. 2020;143(4):1220–32. 10.1093/brain/awaa054. 32206776 10.1093/brain/awaa054 PMC7174054

[5] Mattsson N, Cullen NC, Andreasson U, Zetterberg H, Blennow K. Association between longitudinal plasma neurofilament light and neurodegeneration in patients with Alzheimer disease. JAMA Neurol. 2019;76(7):791–9. 10.1001/jamaneurol.2019.0765. 31009028 10.1001/jamaneurol.2019.0765 PMC6583067

[6] Weston PSJ, Poole T, O’Connor A, et al. Longitudinal measurement of serum neurofilament light in presymptomatic familial Alzheimer’s disease. Alzheimer’s Res Therapy. 2019;11(1):19. 10.1186/s13195-019-0472-5. 10.1186/s13195-019-0472-5 PMC6383280 30786919

[7] Shahim P, Gren M, Liman V, et al. Serum neurofilament light protein predicts clinical outcome in traumatic brain injury. Sci Rep. 2016;6:36791. 10.1038/srep36791. 27819296 10.1038/srep36791 PMC5098187

[8] Teunissen CE, Dijkstra C, Polman C. Biological markers in CSF and blood for axonal degeneration in multiple sclerosis. Lancet Neurol. 2005;4(1):32–41. 10.1016/S1474-4422(04)00964-0. 15620855 10.1016/S1474-4422(04)00964-0

[9] Khalil M, Pirpamer L, Hofer E, et al. Serum neurofilament light levels in normal aging and their association with morphologic brain changes. Nat Commun. 2020;11(1):812. 10.1038/s41467-020-14612-6. 32041951 10.1038/s41467-020-14612-6 PMC7010701

[10] Beydoun MA, Noren Hooten N, Weiss J, et al. Plasma neurofilament light and its association with all-cause mortality risk among urban middle-aged men and women. BMC Med. 2022;20(1):218. 10.1186/s12916-022-02425-x. 35692046 10.1186/s12916-022-02425-x PMC9190073

[11] Rubsamen N, Maceski A, Leppert D, et al. Serum neurofilament light and tau as prognostic markers for all-cause mortality in the elderly general population-an analysis from the MEMO study. BMC Med. 2021;19(1):38. 10.1186/s12916-021-01915-8. 33583409 10.1186/s12916-021-01915-8 PMC7883435

[12] Kaeser SA, Lehallier B, Thinggaard M, Häsler LM, Apel A, Bergmann C, Berdnik D, Jeune B, Christensen K, Grönke S, Partridge L, Wyss-Coray T, Mengel-From J, Jucker M. A neuronal blood marker is associated with mortality in old age. Nat Aging. 2021;1:218–25. 37118632 10.1038/s43587-021-00028-4

[13] Nguyen AD, Malmstrom TK, Aggarwal G, Miller DK, Vellas B, Morley JE. Serum neurofilament light levels are predictive of all-cause mortality in late middle-aged individuals. EBioMedicine. 2022;82:104146. 10.1016/j.ebiom.2022.104146. 35830835 10.1016/j.ebiom.2022.104146 PMC9284367

[14] Manouchehrinia A, Piehl F, Hillert J, et al. Confounding effect of blood volume and body mass index on blood neurofilament light chain levels. Ann Clin Transl Neurol. 2020;7(1):139–43. 10.1002/acn3.50972. 31893563 10.1002/acn3.50972 PMC6952306

[15] Akamine S, Marutani N, Kanayama D, et al. Renal function is associated with blood neurofilament light chain level in older adults. Sci Rep. 2020;10(1):20350. 10.1038/s41598-020-76990-7. 33230211 10.1038/s41598-020-76990-7 PMC7683708

[16] Benkert P, Meier S, Schaedelin S, et al. Serum neurofilament light chain for individual prognostication of disease activity in people with multiple sclerosis: a retrospective modelling and validation study. Lancet Neurol. 2022;21(3):246–57. 10.1016/S1474-4422(22)00009-6. 35182510 10.1016/S1474-4422(22)00009-6

[17] Dittrich A, Ashton NJ, Zetterberg H, et al. Association of chronic kidney disease with plasma NfL and other biomarkers of neurodegeneration: the H70 birth cohort study in Gothenburg. Neurology. 2023;101(3):e277–88. 10.1212/WNL.0000000000207419. 37225431 10.1212/WNL.0000000000207419 PMC10382262

[18] Stocker H, Beyer L, Trares K, et al. Association of kidney function with development of Alzheimer disease and other dementias and dementia-related blood biomarkers. JAMA Netw Open. 2023;6(1):e2252387. 10.1001/jamanetworkopen.2022.52387. 36692879 10.1001/jamanetworkopen.2022.52387 PMC10408272

[19] Zhang B, Zhang C, Wang Y, et al. Effect of renal function on the diagnostic performance of plasma biomarkers for Alzheimer’s disease. Front Aging Neurosci. 2023;15:1150510. 10.3389/fnagi.2023.1150510. 37009461 10.3389/fnagi.2023.1150510 PMC10050758

[20] Beydoun MA, Noren Hooten N, Weiss J, et al. Plasma neurofilament light as blood marker for poor brain white matter integrity among middle-aged urban adults. Neurobiol Aging. 2023;121:52–63. 10.1016/j.neurobiolaging.2022.10.004. 36371816 10.1016/j.neurobiolaging.2022.10.004 PMC9733693

[21] Mielke MM, Syrjanen JA, Blennow K, et al. Plasma and CSF neurofilament light: relation to longitudinal neuroimaging and cognitive measures. Neurology. 2019;93(3):e252–60. 10.1212/WNL.0000000000007767. 31182505 10.1212/WNL.0000000000007767 PMC6656645

[22] Nyberg L, Lundquist A, Nordin Adolfsson A, et al. Elevated plasma neurofilament light in aging reflects brain white-matter alterations but does not predict cognitive decline or Alzheimer’s disease. Alzheimers Dement (Amst). 2020;12(1):e12050. 10.1002/dad2.12050. 32587884 10.1002/dad2.12050 PMC7311800

[23] Beydoun MA, Beydoun HA, Gamaldo AA, Teel A, Zonderman AB, Wang Y. Epidemiologic studies of modifiable factors associated with cognition and dementia: systematic review and meta-analysis. BMC Public Health. 2014;14:643. 10.1186/1471-2458-14-643. 24962204 10.1186/1471-2458-14-643 PMC4099157

[24] Beydoun MA, Shaked D, Hossain S, et al. Vitamin D, folate, and cobalamin serum concentrations are related to brain volume and white matter integrity in urban adults. Front Aging Neurosci. 2020;12:140. 10.3389/fnagi.2020.00140. 32523528 10.3389/fnagi.2020.00140 PMC7261885

[25] Wassenaar TM, Yaffe K, van der Werf YD, Sexton CE. Associations between modifiable risk factors and white matter of the aging brain: insights from diffusion tensor imaging studies. Neurobiol Aging. 2019;80:56–70. 10.1016/j.neurobiolaging.2019.04.006. 31103633 10.1016/j.neurobiolaging.2019.04.006 PMC6683729

[26] Chakravorty S, Jackson N, Chaudhary N, et al. Daytime sleepiness: associations with alcohol use and sleep duration in americans. Sleep Disord. 2014;2014:959152. 10.1155/2014/959152. 24672731 10.1155/2014/959152 PMC3927862

[27] Shapiro AL, Culp S, Azulay Chertok IR. OSA symptoms associated with and predictive of anxiety in middle-aged men: secondary analysis of NHANES data. Arch Psychiatr Nurs. 2014;28(3):200–5. 10.1016/j.apnu.2014.02.002. 24856274 10.1016/j.apnu.2014.02.002

[28] Centers of Disease Control and Prevention. NHNES2007–2008 Overview. https://wwwn.cdc.gov/nchs/nhanes/ContinuousNhanes/overview.aspx?BeginYear=2007. Accessed March 10 2020.

[29] Centers of Disease Control and Prevention. NHANES 2005–2006 Overview. https://wwwn.cdc.gov/nchs/nhanes/ContinuousNhanes/overview.aspx?BeginYear=2005. Accessed March 10 2020.

[30] Beydoun HA, Huang S, Beydoun MA, Hossain S, Zonderman AB. Mediating-moderating effect of allostatic load on the association between dietary approaches to stop hypertension diet and all-cause and cause-specific mortality: 2001–2010 National Health and Nutrition Examination Surveys. Nutrients. 2019. 10.3390/nu11102311. 31569527 10.3390/nu11102311 PMC6836046

[31] Beydoun MA, Beydoun HA, Mode N, et al. Racial disparities in adult all-cause and cause-specific mortality among us adults: mediating and moderating factors. BMC Public Health. 2016;16(1):1113. 10.1186/s12889-016-3744-z. 27770781 10.1186/s12889-016-3744-z PMC5075398

[32] Committee IR. Guidelines for data processing and analysis of the International Physical Activity Questionnaire (IPAQ)—short and long forms. The International Physical Activity Questionnaire (2005).

[33] Raper N, Perloff B, Ingwersen L, Steinfeldt L, Anand J. An overview of USDA’s dietary intake data system. J Food Compos Anal. 2004;17(3–4):545–55. 10.1016/j.jfca.2004.02.013

[34] Mellen PB, Gao SK, Vitolins MZ, Goff DC Jr. Deteriorating dietary habits among adults with hypertension: DASH dietary accordance, NHANES 1988–1994 and 1999–2004. Arch Intern Med. 2008;168(3):308–14. 10.1001/archinternmed.2007.119. 18268173 10.1001/archinternmed.2007.119

[35] US Department of Agriculture ARS, Food Surveys Research Group. . Food and Nutrient Database for Dietary Studies. https://www.ars.usda.gov/northeast-area/beltsville-md-bhnrc/beltsville-human-nutrition-research-center/food-surveys-research-group/docs/fndds-download-databases.

[36] Mellen PB, Gao SK, Vitolins MZ, Goff DC Jr. Deteriorating dietary habits among adults with hypertension: DASH dietary accordance, NHANES 1988–1994 and 1999–2004. Arch Intern Med. 2008;168(3):308–14. 10.1001/archinternmed.2007.119. 18268173 10.1001/archinternmed.2007.119

[37] STATA. Statistics/Data Analysis: Release 16.0. Stata Corporation, Texas (2019).

[38] Discacciati A, Bellavia A, Lee JJ, Mazumdar M, Valeri L. Med4way: a Stata command to investigate mediating and interactive mechanisms using the four-way effect decomposition. Oxford: Oxford University Press; 2019. 10.1093/ije/dyy236 30452641

[39] Discacciati A, Bellavia A, Lee JJ, Mazumdar M, Valeri L. Med4way: a Stata command to investigate mediating and interactive mechanisms using the four-way effect decomposition. Int J Epidemiol. 2018. 10.1093/ije/dyy236. 30452641 10.1093/ije/dyy236

[40] Heckman JJ. Sample selection bias as a specification error. Econometrica. 1979;47:153–61. 10.2307/1912352

[41] Beydoun MA, Noren Hooten N, Beydoun HA, et al. Plasma neurofilament light as a potential biomarker for cognitive decline in a longitudinal study of middle-aged urban adults. Transl Psychiatry. 2021;11(1):436. 10.1038/s41398-021-01563-9. 34420032 10.1038/s41398-021-01563-9 PMC8380245

[42] VanderWeele TJ. Causal interactions in the proportional hazards model. Epidemiology. 2011;22(5):713–7. 10.1097/EDE.0b013e31821db503. 21558856 10.1097/EDE.0b013e31821db503 PMC3150431

[43] Andersson T, Alfredsson L, Kallberg H, Zdravkovic S, Ahlbom A. Calculating measures of biological interaction. Eur J Epidemiol. 2005;20(7):575–9. 10.1007/s10654-005-7835-x. 16119429 10.1007/s10654-005-7835-x

[44] Knol MJ, VanderWeele TJ, Groenwold RH, Klungel OH, Rovers MM, Grobbee DE. Estimating measures of interaction on an additive scale for preventive exposures. Eur J Epidemiol. 2011;26(6):433–8. 10.1007/s10654-011-9554-9. 21344323 10.1007/s10654-011-9554-9 PMC3115067

[45] Pamplin Ii JR, Rudolph KE, Keyes KM, Susser ES, Bates LM. Investigating a paradox: toward a better understanding of the relationships between racial group membership, stress, and major depressive disorder. Am J Epidemiol. 2023;192(11):1845–53. 10.1093/aje/kwad128. 37230957 10.1093/aje/kwad128 PMC11043785

[46] Karriker-Jaffe KJ, Foshee VA, Ennett ST. Examining how neighborhood disadvantage influences trajectories of adolescent violence: a look at social bonding and psychological distress. J Sch Health. 2011;81(12):764–73. 10.1111/j.1746-1561.2011.00656.x. 22070508 10.1111/j.1746-1561.2011.00656.x PMC3499619

[47] Scherling CS, Hall T, Berisha F, et al. Cerebrospinal fluid neurofilament concentration reflects disease severity in frontotemporal degeneration. Ann Neurol. 2014;75(1):116–26. 10.1002/ana.24052. 24242746 10.1002/ana.24052 PMC4020786

[48] Sanchez-Valle R, Heslegrave A, Foiani MS, et al. Serum neurofilament light levels correlate with severity measures and neurodegeneration markers in autosomal dominant Alzheimer’s disease. Alzheimer’s Res Therapy. 2018;10(1):113. 10.1186/s13195-018-0439-y. 10.1186/s13195-018-0439-y PMC6215337 30390718

[49] Weston PSJ, Poole T, Ryan NS, et al. Serum neurofilament light in familial Alzheimer disease: a marker of early neurodegeneration. Neurology. 2017;89(21):2167–75. 10.1212/WNL.0000000000004667. 29070659 10.1212/WNL.0000000000004667 PMC5696646

[50] He L, Morley JE, Aggarwal G, et al. Plasma neurofilament light chain is associated with cognitive decline in non-dementia older adults. Sci Rep. 2021;11(1):13394. 10.1038/s41598-021-91038-0. 34183688 10.1038/s41598-021-91038-0 PMC8238930

[51] Gendron TF, Badi MK, Heckman MG, et al. Plasma neurofilament light predicts mortality in patients with stroke. Sci Transl Med. 2020;12(569):55. 10.1126/scitranslmed.aay1913. 10.1126/scitranslmed.aay1913 PMC9534269 33177179

[52] Uphaus T, Bittner S, Groschel S, et al. NfL (Neurofilament Light Chain) levels as a predictive marker for long-term outcome after ischemic stroke. Stroke. 2019;50(11):3077–84. 10.1161/STROKEAHA.119.026410. 31537188 10.1161/STROKEAHA.119.026410

[53] Staffaroni AM, Kramer AO, Casey M, et al. Association of blood and cerebrospinal fluid tau level and other biomarkers with survival time in Sporadic Creutzfeldt-Jakob disease. JAMA Neurol. 2019;76(8):969–77. 10.1001/jamaneurol.2019.1071. 31058916 10.1001/jamaneurol.2019.1071 PMC6503575

[54] Hviid CVB, Gyldenholm T, Lauridsen SV, Hjort N, Hvas AM, Parkner T. Plasma neurofilament light chain is associated with mortality after spontaneous intracerebral hemorrhage. Clin Chem Lab Med. 2020;58(2):261–7. 10.1515/cclm-2019-0532. 31494627 10.1515/cclm-2019-0532

[55] Hviid CVB, Lauridsen SV, Gyldenholm T, Sunde N, Parkner T, Hvas AM. Plasma neurofilament light chain is associated with poor functional outcome and mortality rate after spontaneous subarachnoid hemorrhage. Transl Stroke Res. 2020;11(4):671–7. 10.1007/s12975-019-00761-4. 31808039 10.1007/s12975-019-00761-4

[56] Korley FK, Goldstick J, Mastali M, et al. Serum NfL (Neurofilament Light Chain) levels and incident stroke in adults with Diabetes Mellitus. Stroke. 2019;50(7):1669–75. 10.1161/STROKEAHA.119.024941. 31138085 10.1161/STROKEAHA.119.024941 PMC6591022

[57] Beydoun MA, Noren Hooten N, Maldonado AI, et al. BMI and allostatic load are directly associated with longitudinal increase in plasma neurofilament light among urban middle-aged adults. J Nutr. 2022;152(2):535–49. 10.1093/jn/nxab381. 34718678 10.1093/jn/nxab381 PMC8826916

[58] Fitzgerald KC, Sotirchos ES, Smith MD, et al. Contributors to serum NfL levels in people without neurologic disease. Ann Neurol. 2022;92(4):688–98. 10.1002/ana.26446. 35730070 10.1002/ana.26446 PMC9489658

[59] Scrimgeour AG, Condlin ML, Loban A, DeMar JC. Omega-3 fatty acids and vitamin D decrease plasma T-Tau, GFAP, and UCH-L1 in experimental traumatic brain injury. Front Nutr. 2021;8:685220. 10.3389/fnut.2021.685220. 34150829 10.3389/fnut.2021.685220 PMC8211733

[60] Hanninen K, Jaaskelainen O, Herukka SK, Soilu-Hanninen M. Vitamin D supplementation and serum neurofilament light chain in interferon-beta-1b-treated MS patients. Brain Behav. 2020;10(9):e01772. 10.1002/brb3.1772. 32705821 10.1002/brb3.1772 PMC7507359

[61] Rossor AM, Reilly MM. Blood biomarkers of peripheral neuropathy. Acta Neurol Scand. 2022;146(4):325–31. 10.1111/ane.13650. 35611606 10.1111/ane.13650 PMC9796925

[62] VanderWeele TJ. A unification of mediation and interaction: a 4-way decomposition. Epidemiology. 2014;25(5):749–61. 10.1097/EDE.0000000000000121. 25000145 10.1097/EDE.0000000000000121 PMC4220271

[63] Woolf SH, Chapman DA, Buchanich JM, Bobby KJ, Zimmerman EB, Blackburn SM. Changes in midlife death rates across racial and ethnic groups in the United States: systematic analysis of vital statistics. BMJ. 2018;362:k3096. 10.1136/bmj.k3096. 30111554 10.1136/bmj.k3096 PMC6092678

