# Supplementary material for: Serum neurofilament light chain as a prognostic marker of all-cause mortality in a national sample of US adults
Source: Eur J Epidemiol. 2024 May 21;39(7):795–809. doi: 10.1007/s10654-024-01131-7 (PMC11343803; doi:10.1007/s10654-024-01131-7)
Supplement: Supplementary file 1 — Supplementary file1 (DOCX 60 KB) [file 10654_2024_1131_MOESM1_ESM.docx]

**ONLINE SUPPLEMENTARY MATERIALS**

**Supplementary methods 1: Nutritional and other blood/urine biomarkers**

Glycated hemoglobin (HbA1c) was measured by ion-exchange high-performance liquid chromatography (HPLC)[ <https://wwwn.cdc.gov/Nchs/Nhanes/2013-2014/GHB_H.htm>]. Total cholesterol was measured by an enzymatic assay, using the Beckman synchron LX20 method [https://wwwn.cdc.gov/nchs/data/nhanes/2013-2014/labmethods/BIOPRO_H_MET_TOTAL_CHOLESTEROL.pdf]. To determine the urinary albumin:creatinine ratio, urinary albumin was measured using a solid-phase fluorescent immunoassay while creatinine is measured by enzymatic method sing a Roche Cobas 6000 analyzer [https://wwwn.cdc.gov/Nchs/Nhanes/2013-2014/ALB_CR_H.htm].

Whole-blood folate was measured by microbiologic assay [https://wwwn.cdc.gov/Nchs/Nhanes/2013-2014/FOLATE_H.htm]. Ultra-high performance liquid chromatography-tandem mass spectrometry (UHPLC-MS/MS) was used to quantify 25-hydroxyvitamin D in the serum [https://wwwn.cdc.gov/Nchs/Nhanes/2013-2014/VID_H.htm]. Serum vitamin B_12_ was measured via ECLIA (electrochemiluminescence)[ https://wwwn.cdc.gov/Nchs/Nhanes/2013-2014/VITB12_H.htm].

**SUPPLEMENTARY TABLE 1. Study sample characteristics by Loge transformed serum NfL category (Below vs. Above median), N=2,071: NHANES 2013-2014**

|  | **Below Median Log_e_ transformed serum NfL** | |  | **Above Median Log_e_ transformed serum NfL** | |  |  |
| --- | --- | --- | --- | --- | --- | --- | --- |
|  | **N=1,046** | |  | **N=1,025** | |  |  |
|  | **Mean/%** | **(SE)** |  | **Mean/%** | **(SE)** |  | **P_NfL_** |
| **Exposures and outcomes** |  |  |  |  |  |  |  |
| NfL, Log_e_ transformed  Mean (SEM) | 2.04 | (0.02) |  | 3.07 | (0.04) |  | <0.001 |
| Died, % | 1.2 | (0.4) |  | 6.0 |  |  | 0.001 |
| **Covariates** |  |  |  |  |  |  |  |
| Age (years): |  |  |  |  |  |  |  |
| Mean (SEM) | 38.5 | (0.6) |  | 52.1 | (0.6) |  | <0.001 |
| % Women | 53.8 | (1.8) |  | 48.7 |  |  | 0.048 |
| Race/Ethnicity: |  |  |  |  | (0.10) |  |  |
| Non-Hispanic White | 58.2 | (4.1) |  | 72.2 | (3.5) |  | __ |
| Non-Hispanic Black | 14.2 | (2.3) |  | 9.7 | (1.0) |  | 0.002 |
| Mexican-American and other Hispanic | 19.0 | (2.7) |  | 11.4 | (2.9) |  | 0.001 |
| Other | 8.6 | (1.1) |  | 6.6 | (1.2) |  | 0.005 |
| Household size | 3.6 | (0.1) |  | 2.84 | (0.07) |  | <0.001 |
| Education: |  |  |  |  |  |  |  |
| < Less Than 9th Grade | 4.1 | (0.8) |  | 4.5 | (0.7) |  | 0.77 |
| 9–11th Grade | 12.3 | (1.0) |  | 10.0 | (2.1) |  | 0.27 |
| High School Grad/GED or Equivalent | 20.3 | (2.3) |  | 20.0 | (1.9) |  | 0.71 |
| Some College or AA Degree | 32.6 | (1.7) |  | 34.5 | (2.2) |  | __ |
| College Graduate or Above | 30.8 | (2.6) |  | 30.6 | (2.9) |  | 0.71 |
| Marital Status: |  |  |  |  |  |  |  |
| Married/Living with Partner | 65.9 | (2.6) |  | 63.8 | (1.7) |  | 0.50 |
| Other | 34.1 | (2.6) |  | 36.2 | (1.7) |  |  |
| Poverty-Income Ratio: |  |  |  |  |  |  |  |
| < 100% | 20.0 | (1.8) |  | 16.2 | (3.3) |  | 0.24 |
| 100%–<200% | 18.6 | (1.2) |  | 20.4 | (2.2) |  | 0.74 |
| ≥ 200% | 61.4 | (2.5) |  | 63.4 | (4.9) |  | __ |
| Smoking Status: |  |  |  |  |  |  |  |
| Never Smoker | 61.4 | (2.1) |  | 51.0 | (3.0) |  | __ |
| Ex-Smoker | 18.7 | (1.8) |  | 26.3 | (2.4) |  | 0.001 |
| Current Smoker | 20.0 | (1.6) |  | 22.7 | (3.4) |  | 0.086 |
| Alcohol Consumption  (≥ 12 glasses in past 12 months): |  |  |  |  |  |  |  |
| Yes | 77.7 | (2.8) |  | 77.5 | (2.6) |  | __ |
| No | 22.3 | (2.8) |  | 22.5 | (2.6) |  | 0.91 |
|  |  |  |  |  |  |  |  |
| Drug ever use, % | 48.9 | (2.6) |  | 42.0 | (2.4) |  | 0.033 |
|  |  |  |  |  |  |  |  |
| Physical Activity,Met.min^-1^/wk | 2,119 | (206.7) |  | 1,994 | (267) |  | 0.59 |
|  |  |  |  |  |  |  |  |
|  |  |  |  |  |  |  |  |
| Body Mass Index *(kg/m^2^)*: |  |  |  |  |  |  |  |
| Mean (SEM) | 29.4 | (0.3) |  | 29.3 | (0.3) |  | 0.87 |
|  |  |  |  |  |  |  |  |
|  |  |  |  |  |  |  |  |
| Systolic Blood Pressure *(mm Hg)*: | 117.0 | (0.5) |  | 122.9 | (0.7) |  | <0.001 |
| Mean (SEM) |  |  |  |  |  |  |  |
|  |  |  |  |  |  |  |  |
|  |  |  |  |  |  |  |  |
|  |  |  |  |  |  |  |  |
| Diastolic Blood Pressure (*mm Hg*):  Mean (SEM) | 68.9 | (0.6) |  | 69.7 | (0.6) |  | 0.24 |
|  |  |  |  |  |  |  |  |
| Total cholesterol, mmol/L | 4.82 | (0.04) |  | 4.97 | (0.05) |  | 0.026 |
| Mean (SEM) |  |  |  |  |  |  |  |
|  |  |  |  |  |  |  |  |
| Glycated Hemoglobin, % | 5.39 | (0.02) |  | 5.83 | (0.04) |  | <0.001 |
|  |  |  |  |  |  |  |  |
|  |  |  |  |  |  |  |  |
| Urinary albumin:creatinine ratio, Log_e_ transformed | 2.06 | (0.04) |  | 2.24 | (0.04) |  | 0.001 |
|  |  |  |  |  |  |  |  |
| Serum vitamin D3, 25(OH)D3, nmol/L | 60.2 | (1.5) |  | 68.6 | (1.46) |  | <0.001 |
|  |  |  |  |  |  |  |  |
| RBC folate, nmol/L | 1,180 | (28.7) |  | 1,312 | (34.6) |  | 0.003 |
|  |  |  |  |  |  |  |  |
| Serum vitamin B-12, pmol/L | 564.0 | (8.6) |  | 643 | (46) |  | 0.11 |
|  |  |  |  |  |  |  |  |
| Self-Rated Health: |  |  |  |  |  |  |  |
| Excellent/Very Good/Good | 85.9 | (1.3) |  | 77.2 | (3.7) |  | __ |
| Fair/Poor | 14.1 | (1.3) |  | 22.8 | (3.6) |  | 0.031 |
|  |  |  |  |  |  |  |  |
| Co-morbidity, % | 8.2 | (1.0) |  | 21.4 | (1.6) |  | <0.001 |
|  |  |  |  |  |  |  |  |
| Energy intake, kcal/d | 2,143 | (32.2) |  | 2,099 | (44) |  | 0.47 |
| DASH Diet Total Score | 2.05 | (0.06) |  | 2.24 | (0.09) |  | 0.095 |
|  |  |  |  |  |  |  |  |
|  |  |  |  |  |  |  |  |
|  |  |  |  |  |  |  |  |

*Abbreviations*: 25(OH)D3=25-hydroxyvitamin D3; ACR=Albumin:Creatinne Ratio; B-12=Vitamin B-12; BMI=Body Mass Index; CDE=Controlled Direct Effect; CHOLESTEROL=Total cholesterol; COMORBID=Co-morbidity Index; DBP=Diastolic Blood Pressure; FOL=Folate; HBA1C=Glycated Hemoglobin; IM=Interaction, mediated; IR=Interaction, Reference; M=Mediators/Effect Modifier; NfL= Plasma Neurofilament Light Chain, Log_e_ transformed; PM-Pure Mediation; RBC=Red Blood Cells; SBP=Systolic Blood Pressure; SRH=Self-rated Health; X=Exposure

**SUPPLEMENTARY TABLE 2A. Association between NfL, potential mediators/moderators and all-cause mortality (Models A-C) , N=2,071: NHANES 2013-2014**

| **Potential Mediators/moderators** |  | **β** | **(SEE)** | **P** |
| --- | --- | --- | --- | --- |
| **BMI** |  |  |  |  |
| Model A: BMI🡪Mortality |  | +0.110 | (0.123) | 0.37 |
| Model B: |  |  |  |  |
| BMI🡪Mortality |  | -0.067 | (0.148) | 0.65 |
| NFL🡪Mortality |  | **+0.644** | **(0.112)** | **<0.001** |
| BMI×NFL🡪Mortality |  | +0.187 | (0.148) | 0.21 |
| Model C: |  |  |  |  |
| BMI🡪NFL |  | -0.013 | (0.020) | 0.49 |
| **SBP** |  |  |  |  |
| Model A: SBP🡪Mortality |  | -0.058 | (0.163) | 0.72 |
| Model B: |  |  |  |  |
| SBP🡪Mortality |  | -0.102 | (0.236) | 0.67 |
| NFL🡪Mortality |  | **+0.663** | **(0.113)** | **<0.001** |
| SBP×NFL🡪Mortality |  | +0.039 | (0.092) | 0.68 |
| Model C: |  |  |  |  |
| SBP🡪NFL |  | **+0.045** | **(0.021)** | **0.034** |
| **DBP** |  |  |  |  |
| Model A: DBP🡪Mortality |  | -0.052 | (0.151) | 0.74 |
| Model B: |  |  |  |  |
| DBP🡪Mortality |  | -0.092 | (0.224) | 0.68 |
| NFL🡪Mortality |  | **+0.666** | **(0.108)** | **<0.001** |
| DBP×NFL🡪Mortality |  | +0.084 | (0.095) | 0.38 |
| Model C: |  |  |  |  |
| DBP🡪NFL |  | -0.005 | (0.020) | 0.81 |
| **HBA1C** |  |  |  |  |
| Model A: HBA1C🡪Mortality |  | **+0.304** | **(0.080)** | **<0.001** |
| Model B: |  |  |  |  |
| HBA1C🡪Mortality |  | -0.002 | (0.126) | 0.99 |
| NFL🡪Mortality |  | **+0.572** | **(0.116)** | **<0.001** |
| HBA1C×NFL🡪Mortality |  | **+0.127** | **(0.052)** | **0.014** |
| Model C: |  |  |  |  |
| HBA1C🡪NFL |  | **+0.142** | **(0.020)** | **<0.001** |
| **CHOLESTEROL** |  |  |  |  |
| Model A: CHOLESTEROL🡪Mortality |  | ***-0.280*** | ***(0.161)*** | ***0.083*** |
| Model B: |  |  |  |  |
| CHOLESTEROL🡪Mortality |  | **-0.416** | **(0.188)** | **0.027** |
| NFL🡪Mortality |  | **+0.716** | **(0.139)** | **<0.001** |
| CHOLESTEROL×NFL🡪Mortality |  | +0.131 | (0.103) | 0.20 |
| Model C: |  |  |  |  |
| CHOLESTEROL🡪NFL |  | -0.025 | (0.019) | 0.20 |
| **ACR** |  |  |  |  |
| Model A: ACR🡪Mortality |  | **+0.475** | **(0.106)** | **<0.001** |
| Model B: |  |  |  |  |
| ACR🡪Mortality |  | +0.231 | (0.181) | 0.20 |
| NFL🡪Mortality |  | **+0.538** | **(0.117)** | **<0.001** |
| ACR×NFL🡪Mortality |  | +0.134 | (0.106) | 0.21 |
| Model C: |  |  |  |  |
| ACR🡪NFL |  | **+0.098** | **(0.021)** | **<0.001** |
| **VITD** |  |  |  |  |
| Model A: VITD🡪Mortality |  | **-0.335** | **(0.145)** | **0.021** |
| Model B: |  |  |  |  |
| VITD🡪Mortality |  | **-0.300** | **(0.152)** | **0.048** |
| NFL🡪Mortality |  | **+0.688** | **(0.118)** | **<0.001** |
| VITD×NFL🡪Mortality |  | -0.031 | (0.077) | 0.69 |
| Model C: |  |  |  |  |
| VITD🡪NFL |  | **+0.057** | **(0.021)** | **0.006** |
| **RBC FOL** |  |  |  |  |
| Model A: RBC FOL🡪Mortality |  | +0.118 | (0.094) | 0.21 |
| Model B: |  |  |  |  |
| RBC FOL🡪Mortality |  | +0.160 | (0.100) | 0.11 |
| NFL🡪Mortality |  | **+0.706** | **(0.110)** | **<0.001** |
| RBC FOL×NFL🡪Mortality |  | -0.129 | (0.089) | 0.15 |
| Model C: |  |  |  |  |
| RBC FOL🡪NFL |  | **+0.050** | **(0.020)** | **0.013** |
| **B-12** |  |  |  |  |
| Model A: B-12🡪Mortality |  | **+0.216** | **(0.076)** | **0.004** |
| Model B: |  |  |  |  |
| B-12🡪Mortality |  | +0.022 | (0.084) | 0.79 |
| NFL🡪Mortality |  | **+0.586** | **(0.124)** | **<0.001** |
| B-12×NFL🡪Mortality |  | **+0.173** | **(0.065)** | **0.008** |
| Model C: |  |  |  |  |
| B-12🡪NFL |  | +0.022 | (0.019) | 0.23 |
| **COMORBID** |  |  |  |  |
| Model A: COMORBID🡪Mortality |  | +0.423 | (0.295) | 0.15 |
| Model B: |  |  |  |  |
| COMORBID🡪Mortality |  | **+1.007** | **(0.367)** | **0.006** |
| NFL🡪Mortality |  | **+0.861** | **(0.140)** | **<0.001** |
| COMORBID×NFL🡪Mortality |  | **-0.601** | **(0.217)** | **0.006** |
| Model C: |  |  |  |  |
| COMORBID🡪NFL |  | +0.095 | (0.058) | 0.10 |
| **SRH** |  |  |  |  |
| Model A: SRH🡪Mortality |  | **+1.144** | **(0.315)** | **<0.001** |
| Model B: |  |  |  |  |
| SRH🡪Mortality |  | ***+0.726*** | ***(0.403)*** | ***0.072*** |
| NFL🡪Mortality |  | **+0.498** | **(0.148)** | **0.001** |
| SRH×NFL🡪Mortality |  | +0.183 | (0.194) | 0.35 |
| Model C: |  |  |  |  |
| SRH🡪NFL |  | **+0.175** | **(0.048)** | **<0.001** |

*Abbreviations*: ACR=Albumin:Creatinne Ratio; B-12=Vitamin B-12; BMI=Body Mass Index; CHOLESTEROL=Total cholesterol; COMORBID=Co-morbidity Index; DBP=Diastolic Blood Pressure; FOL=Folate; HBA1C=Glycated Hemoglobin; M=Mediators/Effect Modifier; NfL= Plasma Neurofilament Light Chain, Log_e_ transformed; RBC=Red Blood Cells; SBP=Systolic Blood Pressure; SRH=Self-rated Health; VITD= Serum Vitamin D3 or 25(OH)D3; X=Exposure

*Notes*: All models adjusted for exogenous covariates. Those included exogenous covariates included age, sex, race/ethnicity, PIR, education, smoking, ever drug use, alcohol use, DASH, total caloric intake, physical activity, household size and marital status. Models A and B are a series of Cox PH models with main predictor being each potential mediator/moderator and NfL exposure for Model B. Model B included main effects of NfL and interaction between NfL and each potential mediator/moderator. Model C are a series of linear regression models between NfL, the main outcome, and each potential mediator/moderator as the main predictor. Only main effects are included in Model C. NfL and all continuous potential mediators/moderators were standardized z-scored. NfL and ACR were Log_e_ transformed. COMORBID and SRH are binary variables.

**SUPPLEMENTARY TABLE 2B. Association between NfL, Log_e_ transformed (above vs. below median), potential moderators (binary or above vs. below median) and all-cause mortality (Model B: interaction on additive scale), N=2,071: NHANES 2013-2014**

|  |  |  |  | **ERR_01_** | **ERR_10_** | **ERR_11_** | **RERI** | **AP** | **SI** |
| --- | --- | --- | --- | --- | --- | --- | --- | --- | --- |
| **Potential**  **Moderator, binary** | **Exposure,**  **binary** | **Imputation** |  | **β±SE_rob_** | **β±SE_rob_** | **β±SE_rob_** | **β±SE_rob_** | **β±SE_rob_** | **β±SE_rob_** |
| BMI | NfL | 1 |  | +0.88±1.19 | +1.59±1.45 | **+2.65±2.06*** | +0.19±1.17 | +0.05±0.32 | +1.08±0.52 |
| BMI | NfL | 2 |  | +0.87±1.18 | +1.61±1.47 | **+2.73±2.09*** | +0.25±1.20 | +0.07±0.32 | +1.10±0.54 |
| BMI | NfL | 3 |  | +0.88±1.17 | +1.47±1.37 | **+2.76±2.08*** | +0.42±1.15 | +0.11±0.31 | +1.17±0.59 |
| BMI | NfL | 4 |  | +0.82±1.15 | +1.37±1.34 | **+2.81±2.12*** | +0.63±1.14 | +0.17±0.30 | +1.30±0.70 |
| BMI | NfL | 5 |  | +0.83±1.15 | +1.42±1.39 | **+2.76±2.10*** | +0.51±1.17 | +0.14±0.31 | +1.23±0.66 |
|  |  |  |  |  |  |  |  |  |  |
| SBP | NfL | 1 |  | +0.03±0.61 | +1.59±1.43 | +1.01±1.07 | -0.61±1.10 | -0.30±0.49 | +0.63±0.37 |
| SBP | NfL | 2 |  | +0.17±0.69 | +1.91±1.64 | +1.14±1.17 | -0.94±1.30 | -0.44±0.51 | +0.55±0.29 |
| SBP | NfL | 3 |  | -0.21±0.45 | +1.39±1.23 | +0.67±0.84 | -0.51±0.94 | -0.31±0.51 | +0.57±0.41 |
| SBP | NfL | 4 |  | -0.21±0.47 | +1.31±1.20 | +0.75±0.88 | -0.35±0.92 | -0.20±0.49 | +0.68±0.50 |
| SBP | NfL | 5 |  | +0.02±0.60 | +1.55±1.39 | +1.03±1.09 | -0.54±1.07 | -0.27±0.48 | +0.65±0.40 |
|  |  |  |  |  |  |  |  |  |  |
| DBP | NfL | 1 |  | -0.27±0.47 | +0.93±0.99 | +0.81±0.87 | +0.16±0.72 | +0.08±0.41 | +1.24±1.49 |
| DBP | NfL | 2 |  | -0.53±0.31 | +0.74±0.82 | +0.47±0.67 | +0.26±0.61 | +0.18±0.44 | +2.22±8.09 |
| DBP | NfL | 3 |  | -0.61±0.26 | +0.52±0.72 | +0.33±0.60 | +0.42±0.50 | +0.32±0.43 | -3.65±… |
| DBP | NfL | 4 |  | -0.46±0.34 | +0.84±0.89 | +0.50±0.69 | +0.11±0.69 | +0.07±0.47 | +1.28±2.48 |
| DBP | NfL | 5 |  | -0.22±0.48 | +0.68±0.90 | +1.15±1.06 | +0.69±0.67 | +0.32±0.35 | +2.50±4.92 |
|  |  |  |  |  |  |  |  |  |  |
| HBA1C | NfL | 1 |  | +0.64±1.11 | +1.38±1.47 | +2.65±2.10 | +0.24±1.07 | +0.07±0.33 | +1.12±0.61 |
| HBA1C | NfL | 2 |  | +0.63±1.10 | +1.38±1.46 | +2.37±2.17 | +0.37±1.08 | +0.11±0.33 | +1.18±0.66 |
| HBA1C | NfL | 3 |  | +0.74±1.21 | +1.51±1.66 | +2.46±2.37 | +0.20±1.14 | +0.06±0.33 | +1.09±0.56 |
| HBA1C | NfL | 4 |  | +0.72±1.16 | +1.38±1.47 | +2.49±2.24 | +0.39±1.11 | +0.11±0.32 | +1.18±0.65 |
| HBA1C | NfL | 5 |  | +0.74±1.24 | +1.51±1.68 | +2.48±2.42 | +0.23±1.14 | +0.07±0.34 | +1.10±0.59 |
|  |  |  |  |  |  |  |  |  |  |
| CHOLESTEROL | NfL | 1 |  | -0.62±0.25 | +0.52±0.73 | +0.21±0.58 | +0.32±0.55 | +0.26±0.50 | -2.07±… |
| CHOLESTEROL | NfL | 2 |  | -0.63±0.25 | +0.55±0.75 | +0.21±0.55 | +0.30±0.56 | +0.24±0.50 | -2.58±… |
| CHOLESTEROL | NfL | 3 |  | -0.63±0.25 | +0.52±0.74 | +0.21±0.55 | +0.32±0.56 | +0.26±0.50 | -1.97±… |
| CHOLESTEROL | NfL | 4 |  | -0.63±0.24 | +0.59±0.76 | +0.17±0.54 | +0.21±0.59 | +0.18±0.53 | -4.48±… |
| CHOLESTEROL | NfL | 5 |  | -0.63±0.25 | +0.58±0.76 | +0.18±0.54 | +0.22±0.59 | +0.19±0.52 | -4.49±… |
|  |  |  |  |  |  |  |  |  |  |
| ACR | NfL | 1 |  | +0.09±0.64 | +0.21±0.65 | **+2.45±1.82*** | +2.15±1.16 | **+0.63±0.21**** | +8.4±28.0 |
| ACR | NfL | 2 |  | +0.06±0.64 | +0.29±0.69 | **+2.38±1.79*** | +2.03±1.11 | **+0.60±0.22**** | +6.8±19.0 |
| ACR | NfL | 3 |  | +0.08±0.65 | +0.27±0.68 | **+2.42±1.81*** | +2.07±1.14 | **+0.60±0.21**** | +6.8±18.5 |
| ACR | NfL | 4 |  | +0.11±0.67 | +0.34±0.71 | **+2.40±1.81*** | +1.94±1.13 | **+0.57±0.22*** | +5.4±11.5 |
| ACR | NfL | 5 |  | +0.12±0.68 | +0.25±0.69 | **+2.56±1.89*** | +2.19±1.22 | **+0.61±0.22**** | +6.8±17.8 |
|  |  |  |  |  |  |  |  |  |  |
| VITD | NfL | 1 |  | -0.15±0.56 | **+2.17±1.54*** | +0.15±0.61 | -1.88±1.45 | -1.63±0.90 | +0.07±0.24 |
| VITD | NfL | 2 |  | -0.12±0.58 | **+2.21±1.56*** | +0.19±0.63 | -1.90±1.47 | -1.60±0.88 | +0.09±0.23 |
| VITD | NfL | 3 |  | -0.09±0.59 | **+2.22±1.57*** | +0.20±0.63 | -1.93±1.48 | -1.62±0.88 | +0.09±0.23 |
| VITD | NfL | 4 |  | -0.11±0.58 | **+2.26±1.58*** | +0.16±0.61 | -1.99±1.49 | -1.71±0.91 | +0.08±0.23 |
| VITD | NfL | 5 |  | -0.12±0.59 | **+2.27±1.59*** | +0.18±0.62 | -1.97±1.50 | -1.67±0.90 | +0.08±0.23 |
|  |  |  |  |  |  |  |  |  |  |
| RBC FOL | NfL | 1 |  | +0.74±1.03 | **+1.90±1.49*** | **+2.22±1.59*** | -0.41±1.34 | -0.13±0.41 | +0.84±0.42 |
| RBC FOL | NfL | 2 |  | +0.70±1.00 | **+1.86±1.47*** | **+2.28±1.62*** | -0.28±1.31 | -0.085±0.39 | +0.89±0.44 |
| RBC FOL | NfL | 3 |  | +0.74±1.00 | **+1.87±1.46*** | **+2.26±1.59*** | -0.36±1.32 | -0.11±0.40 | +0.86±0.43 |
| RBC FOL | NfL | 4 |  | +0.65±0.96 | **+1.79±1.43*** | **+2.22±1.56*** | -0.22±1.27 | -0.07±0.39 | +0.90±0.47 |
| RBC FOL | NfL | 5 |  | +0.73±1.03 | **+1.89±1.49*** | **+2.23±1.59*** | -0.39±1.35 | -0.12±0.41 | +0.85±0.43 |
|  |  |  |  |  |  |  |  |  |  |
| B-12 | NfL | 1 |  | +0.20±0.75 | +1.37±1.21 | +1.39±1.28 | -0.18±1.00 | -0.08±0.41 | +0.88±0.55 |
| B-12 | NfL | 2 |  | +0.20±0.76 | +1.46±1.25 | +1.37±1.27 | -0.29±1.01 | -0.12±0.42 | +0.83±0.50 |
| B-12 | NfL | 3 |  | +0.26±0.78 | +1.43±1.24 | +1.42±1.30 | -0.26±1.02 | -0.11±0.41 | +0.85±0.50 |
| B-12 | NfL | 4 |  | +0.22±0.76 | +1.45±1.24 | +1.39±1.29 | -0.28±1.02 | -0.12±0.42 | +0.82±0.50 |
| B-12 | NfL | 5 |  | +0.20±0.76 | +1.40±1.23 | +1.40±1.29 | -0.20±1.03 | -0.08±0.42 | +0.88±0.55 |
|  |  |  |  |  |  |  |  |  |  |
| COMORBID | NfL | 1 |  | +2.27±2.32 | **+1.75±1.20*** | **+2.61±1.74**** | -1.41±2.32 | -0.40±0.64 | +0.65±0.38 |
| COMORBID | NfL | 2 |  | +2.45±2.44 | **+1.84±1.23*** | **+2.66±1.77**** | -1.62±2.45 | -0.44±0.67 | +0.62±0.37 |
| COMORBID | NfL | 3 |  | +2.48±2.47 | **+1.80±1.21*** | **+2.58±1.75**** | -1.70±2.48 | -0.47±0.70 | +0.60±0.36 |
| COMORBID | NfL | 4 |  | +2.36±2.37 | **+1.79±1.21*** | **+2.65±1.75**** | -1.50±2.39 | -0.41±0.66 | +0.64±0.38 |
| COMORBID | NfL | 5 |  | +2.31±2.36 | **+1.76±1.20*** | **+2.67±1.76**** | -1.40±2.37 | -0.38±0.65 | +0.66±0.39 |
|  |  |  |  |  |  |  |  |  |  |
| SRH | NfL | 1 |  | +2.47±2.28 | +0.98±0.99 | **+4.73±3.25**** | +1.28±2.04 | +0.22±0.32 | +1.37±0.73 |
| SRH | NfL | 2 |  | +2.42±2.23 | +1.02±1.01 | **+4.85±3.29**** | +1.41±2.03 | +0.24±0.31 | +1.41±0.74 |
| SRH | NfL | 3 |  | +2.51±2.30 | +1.06±1.03 | **+4.84±3.28**** | +1.28±2.07 | +0.22±0.32 | +1.36±0.71 |
| SRH | NfL | 4 |  | +1.90±2.00 | +0.94±0.99 | **+4.89±3.30**** | +2.06±1.97 | +0.35±0.28 | +1.72±1.00 |
| SRH | NfL | 5 |  | +1.70±1.77 | +0.84±0.88 | **+4.47±2.95**** | +1.93±1.92 | +0.35±0.28 | +1.76±1.07 |

*Abbreviations*: ACR=Albumin:Creatinne Ratio; AP=Attributable Proportion; B-12=Vitamin B-12; BMI=Body Mass Index; CHOLESTEROL=Total cholesterol; COMORBID=Co-morbidity Index; DBP=Diastolic Blood Pressure; ERR=Excess Relative Risk; FOL=Folate; HBA1C=Glycated Hemoglobin; M=Mediators/Effect Modifier; NfL= Plasma Neurofilament Light Chain, Log_e_ transformed; RBC=Red Blood Cells; RERI=Relative Excess Risk due to Interaction; SBP=Systolic Blood Pressure; SE_rob_=robust standard error; SI=Synergy Index; SRH=Self-rated Health; VITD= Serum Vitamin D3 or 25(OH)D3; X=Exposure

*Notes*: All models adjusted for exogenous covariates. Those included exogenous covariates included age, sex, race/ethnicity, PIR, education, smoking, ever drug use, alcohol use, DASH, total caloric intake, physical activity, household size and marital status. The model presented in this part of the Table included main effects of NfL and interaction between NfL and each potential mediator/moderator and main effect of the potential mediator/moderator. NfL and all continuous potential mediators/moderators are binary variables, with originally continuous variables transformed into 0=below median and 1=above median. NfL and ACR were Log_e_ transformed. BMI through B-12 were transformed into “above median”=1 vs. “below median”=0; whereas COMORBID and SRH were binary variables. P>|z| for synergy index (SI) is for test H0: SI=1. Robust standard errors are computed for the Hazard Ratios (HR), not the ERRs. HR=ERR+1. See Methods section for details.

*P<0.05; **P<0.010; ***P<0.001
